# Supplementary material for: Testing the role of online group-based supervision for local humanitarian workers following a crisis: A mixed-methods longitudinal study
Source: PLOS Glob Public Health. 2025 Aug 18;5(8):e0004635. doi: 10.1371/journal.pgph.0004635 (PMC12360539; doi:10.1371/journal.pgph.0004635)
Supplement: S1 Text — (PDF) [file pgph.0004635.s001.pdf]

Mplus Code for Multiple Imputation

```
Mplus VERSION 8.10
MUTHEN & MUTHEN
INPUT INSTRUCTIONS
TITLE: imputation
DATA: file is data1.dat;
Variable: names =
ID
org
age
gender
trauma
c_win1

c_win2
c_win3
c_win4
c_win5
c_win6
c_win7
c_win8
c_win9
c_win10
c_win13
c_win14
c_win15
c_win16
c_win17
c_win18
c_win19
```

c_win20

c_win21

c_win22

c_win23

c_win24

c_win25

c_win26

k61

k62

k63

k64

k65

k66

k67

k68

k69

k610

k613

k614

k615

k616

k617

k618

k619

k620

k621

k622

k623

k624

k625

k626

ind_s1

ind_s2

ind_s3

ind_s4

ind_s5

ind_s6

ind_s7

ind_s8

ind_s9

ind_s10

ind_s13

ind_s14

ind_s15

ind_s16

ind_s17

ind_s18

ind_s19

ind_s20

ind_s21

ind_s22

ind_s23

ind_s24

ind_s25

ind_s26

gr_s1

gr_s2

gr_s3

gr_s4

gr_s5

```
gr_s6
gr_s7
gr_s8
gr_s9
gr_s10
gr_s13
gr_s14
gr_s15
gr_s16
gr_s17
gr_s18
gr_s19
gr_s20
gr_s21
gr_s22
gr_s23
gr_s24
gr_s25
gr_s26
;
usevariables = ID
org
age
gender
trauma
c_win1
c_win2
c_win3
c_win4
c_win5
```

c_win6

c_win7

c_win8

c_win9

c_win10

c_win13

c_win14

c_win15

c_win16

c_win17

c_win18

c_win19

c_win20

c_win21

c_win22

c_win23

c_win24

c_win25

c_win26

k61

k62

k63

k64

k65

k66

k67

k68

k69

k610

k613

```
k614
k615
k616
k617
k618
k619
k620
k621
k622
k623
k624
k625
k626
;
MISSING = ALL (-999);
DATA IMPUTATION:
IMPUTE = c_win1
c_win2
c_win3
c_win4
c_win5
c_win6
c_win7
c_win8
c_win9
c_win10
c_win13
c_win14
c_win15
c_win16
```

c_win17

c_win18

c_win19

c_win20

c_win21

c_win22

c_win23

c_win24

c_win25

c_win26

k61

k62

k63

k64

k65

k66

k67

k68

k69

k610

k613

k614

k615

k616

k617

k618

k619

k620

k621

k622

```
k623
k624
k625
k626
;
NDATASETS = 10;
SAVE = imputen*.dat;
MODEL = regression;
VALUES =
c_win1 (30-45)
c_win2 (30-44)
c_win3 (29-45)
c_win4 (23-45)
c_win5 (24-45)
c_win6 (24-44)
c_win7 (23-45)
c_win8 (24-45)
c_win9 (23-45)
c_win10 (23-45)
c_win13 (23-45)
c_win14 (23-45)
c_win15 (23-45)
c_win16 (23-45)
c_win17 (23-45)
c_win18 (23-45)
c_win19 (23-45)
c_win20 (24-45)
c_win21 (25-45)
c_win22 (23-45)
c_win23 (23-45)
```

c_win24 (24-45)

c_win25 (23-45)

c_win26 (26-45)

k61 (1-15)

k62 (0-14)

k63 (0-14)

k64 (0-17)

k65 (0-18)

k66 (0-18)

k67 (0-18)

k68 (0-18)

k69 (0-18)

k610 (0-18)

k613 (0-18)

k614 (0-18)

k615 (0-14)

k616 (0-18)

k617 (0-18)

k618 (0-18)

k619 (0-18)

k620 (0-17)

k621 (0-14)

k622 (0-17)

k623 (0-18)

k624 (0-18)

k625 (0-15)

k626 (0-15)

;

ROUNDING =

c_win1 (0)

c_win2 (0)

c_win3 (0)

c_win4 (0)

c_win5 (0)

c_win6 (0)

c_win7 (0)

c_win8 (0)

c_win9 (0)

c_win10 (0)

c_win13 (0)

c_win14 (0)

c_win15 (0)

c_win16 (0)

c_win17 (0)

c_win18 (0)

c_win19 (0)

c_win20 (0)

c_win21 (0)

c_win22 (0)

c_win23 (0)

c_win24 (0)

c_win25 (0)

c_win26 (0)

k61 (0)

k62 (0)

k63 (0)

k64 (0)

k65 (0)

k66 (0)

k67 (0)

k68 (0)

k69 (0)

k610 (0)

k613 (0)

k614 (0)

k615 (0)

k616 (0)

k617 (0)

k618 (0)

k619 (0)

k620 (0)

k621 (0)

k622 (0)

k623 (0)

k624 (0)

k625 (0)

k626 (0);

ANALYSIS: TYPE = BASIC;

OUTPUT: TECH8;
