# Supplementary material for: Testing the role of online group-based supervision for local humanitarian workers following a crisis: A mixed-methods longitudinal study
Source: PLOS Glob Public Health. 2025 Aug 18;5(8):e0004635. doi: 10.1371/journal.pgph.0004635 (PMC12360539; doi:10.1371/journal.pgph.0004635)
Supplement: S2 Text — (PDF) [file pgph.0004635.s002.pdf]

R code for Bayesian hierarchical model estimation

Run the model with observed data for the outcome variable K6

```{r}
data_pw$ID_new <- as.factor(data_pw$ID_new)

# Load the brms package
library(brms)

 priors_obs <- c(
  set_prior("normal(0, 0.2)", class = "b", coef = "time_short"),
  set_prior("normal(0, 0.2)", class = "b", coef = "AC_slope_short"),#expect no change and small
SD given validated scale and most things staying constant
  set_prior("normal(0, 0.2)", class = "b", coef = "S_B_E_slope_short"),
  set_prior("normal(2, 0.2)", class = "b", coef = "earth"),#expect big jump - nagamine after earth
was 11
  set_prior("normal(-0.2, 0.2)", class = "b", coef = "sae_k"),#expect small improvement after
initial bump after earthquake
  set_prior("normal(6.67, 4.6)", class = "Intercept") #from Foo et al.
)


# Define the model using brms
model_earth_brms_noT <- brm(k6_winsor ~ time_short + AC_slope_short +
S_B_E_slope_short + earth + sae_k
                        + (1 | ID_new),
                     data = data_pw,
                     family = gaussian(),
                      prior = priors_obs,
                     chains = 4,
                     cores = 4,
                     iter = 15000)
```

RUN IT AGAIN with the imputed data comparing prior settings

Run with Strongly informative priors

```{r}
    # Define prior settings
 strongly_informative_priors <- c(
  set_prior("normal(0, 0.2)", class = "b", coef = "time_short"),
  set_prior("normal(0, 0.2)", class = "b", coef = "AC_slope_short"),#expect no change and small
SD given validated scale and most things staying constant
  set_prior("normal(0, 0.2)", class = "b", coef = "S_B_E_slope_short"),
```

````
  set_prior("normal(2, 0.2)", class = "b", coef = "earth"),#expect big jump - nagamine after earth was 11
  set_prior("normal(-0.2, 0.2)", class = "b", coef = "sae_k"),#expect small improvement after initial bump after earthquake
  set_prior("normal(6.67, 4.6)", class = "Intercept") #from Foo et al.
)

    # Fit models for moderate prior setting
  strong_prior_model <- brm_multiple(
    formula = k6 ~ time_short + AC_slope_short + S_B_E_slope_short  + earth  + sae_k +  (1 | ID),
    family = gaussian(),
    data = mids_object,
    prior = strongly_informative_priors ,
    seed = 112,
    chains = 4,
    cores=4,
    iter = 15000,
  )
```
    ```{r}
    # Define prior settings
moderately_informative_priors <- c(
  set_prior("normal(0, 1)", class = "b", coef = "time_short"),
  set_prior("normal(0, 1)", class = "b", coef = "AC_slope_short"),
  set_prior("normal(0, 1)", class = "b", coef = "S_B_E_slope_short"),
  set_prior("normal(2, 1)", class = "b", coef = "earth"),
  set_prior("normal(-0.2, 1)", class = "b", coef = "sae_k"),
  set_prior("normal(6.67, 5)", class = "Intercept") # Adjusted for moderate uncertainty
    )
    # Fit models for moderate prior setting
  moderate_prior_model <- brm_multiple(
    formula = k6 ~ time_short + AC_slope_short + S_B_E_slope_short  + earth  + sae_k +  (1 | ID),
    family = gaussian(),
    data = mids_object,
    prior = moderately_informative_priors ,
    seed = 112,
    chains = 4,
    cores =8,
    iter = 15000,
  )
```
    Weakly informative Priors model
    ```{r}
library(rstan)
````

````r
library(brms)
library(parallel)
rstan_options(auto_write = TRUE)
options(mc.cores = 4)

    # Define prior settings
weakly_informative_priors <- c(
  set_prior("normal(0, 10)", class = "b", coef = "time_short"),
  set_prior("normal(0, 10)", class = "b", coef = "AC_slope_short"),
  set_prior("normal(0, 10)", class = "b", coef = "S_B_E_slope_short"),
  set_prior("normal(2, 10)", class = "b", coef = "earth"),
  set_prior("normal(-0.2, 1)", class = "b", coef = "sae_k"),
  set_prior("normal(6.67, 5)", class = "Intercept") # Adjusted for moderate uncertainty
    )
# Fit models for weak prior setting

  weak_prior_model <- brm_multiple(
    formula = k6 ~ time_short + AC_slope_short + S_B_E_slope_short  + earth  + sae_k +  (1 |
ID),
    family = gaussian(),
    data = mids_object,
    prior = weakly_informative_priors ,
    seed = 112,
    chains = 4,
    cores = 8,
    iter = 15000,
  )
```
````
````
