# Supplementary material for: Testing the role of online group-based supervision for local humanitarian workers following a crisis: A mixed-methods longitudinal study
Source: PLOS Glob Public Health. 2025 Aug 18;5(8):e0004635. doi: 10.1371/journal.pgph.0004635 (PMC12360539; doi:10.1371/journal.pgph.0004635)
Supplement: S3 Text — (PDF) [file pgph.0004635.s003.pdf]

model_outputs_k6

```
library(brms)
```

```
## Warning: package 'brms' was built under R version 4.4.1
```

```
## Loading required package: Rcpp
```

```
## Loading 'brms' package (version 2.21.0). Useful instructions
## can be found by typing help('brms'). A more detailed introduction
## to the package is available through vignette('brms_overview').
```

```
##
## Attaching package: 'brms'
```

```
## The following object is masked from 'package:stats':
##
##     ar
```

```
model_earth_brms_noT <- readRDS("model_earth_brms_noTk.rds")

# Print the summary of the model
summary(model_earth_brms_noT)
```

```
##  Family: gaussian
##   Links: mu = identity; sigma = identity
## Formula: k6_winsor ~ time_short + AC_slope_short + S_B_E_slope_short + earth + sae_k + (1
| ID_new)
##    Data: data_pw (Number of observations: 670)
##   Draws: 4 chains, each with iter = 15000; warmup = 7500; thin = 1;
##          total post-warmup draws = 30000
##
## Multilevel Hyperparameters:
## ~ID_new (Number of levels: 55)
##               Estimate Est.Error l-95% CI u-95% CI Rhat Bulk_ESS Tail_ESS
## sd(Intercept)     3.28      0.35     2.67     4.02 1.00     5442    10933
##
## Regression Coefficients:
##                   Estimate Est.Error l-95% CI u-95% CI Rhat Bulk_ESS Tail_ESS
## Intercept             5.62      0.57     4.48     6.73 1.00     5317    11100
## time_short            0.08      0.04    -0.01     0.17 1.00    23412    22497
## AC_slope_short        0.00      0.07    -0.13     0.14 1.00    39008    22960
## S_B_E_slope_short     0.05      0.13    -0.21     0.31 1.00    32535    24847
## earth                 1.95      0.19     1.59     2.32 1.00    46462    23131
## sae_k                -0.10      0.11    -0.33     0.12 1.00    22307    22869
##
## Further Distributional Parameters:
##       Estimate Est.Error l-95% CI u-95% CI Rhat Bulk_ESS Tail_ESS
## sigma     3.18      0.09     3.01     3.37 1.00    39635    22794
##
## Draws were sampled using sampling(NUTS). For each parameter, Bulk_ESS
## and Tail_ESS are effective sample size measures, and Rhat is the potential
## scale reduction factor on split chains (at convergence, Rhat = 1).
```

```
# Test if the coefficient of AC_slope is equal to the coefficient of sae_k
hypothesis3 <- hypothesis(model_earth_brms_noT, "AC_slope_short = sae_k")

# Display the results
print(hypothesis3)
```

```
## Hypothesis Tests for class b:
##                 Hypothesis Estimate Est.Error CI.Lower CI.Upper Evid.Ratio
## 1 (AC_slope_short)-... = 0      0.1      0.12    -0.14     0.34         NA
##   Post.Prob Star
## 1        NA
## ---
## 'CI': 90%-CI for one-sided and 95%-CI for two-sided hypotheses.
## '*': For one-sided hypotheses, the posterior probability exceeds 95%;
## for two-sided hypotheses, the value tested against lies outside the 95%-CI.
## Posterior probabilities of point hypotheses assume equal prior probabilities.
```

```
# Test if the coefficient of AC_slope is equal to the coefficient of S_B_E_slope
hypothesis4 <- hypothesis(model_earth_brms_noT, "AC_slope_short = S_B_E_slope_short")

# Display the results
print(hypothesis4)
```

```
## Hypothesis Tests for class b:
##                  Hypothesis Estimate Est.Error CI.Lower CI.Upper Evid.Ratio
## 1 (AC_slope_short)-... = 0    -0.04      0.13     -0.3     0.21         NA
##   Post.Prob Star
## 1        NA
## ---
## 'CI': 90%-CI for one-sided and 95%-CI for two-sided hypotheses.
## '*': For one-sided hypotheses, the posterior probability exceeds 95%;
## for two-sided hypotheses, the value tested against lies outside the 95%-CI.
## Posterior probabilities of point hypotheses assume equal prior probabilities.
```

```
pp_check(model_earth_brms_noT, ndraws=100)
```

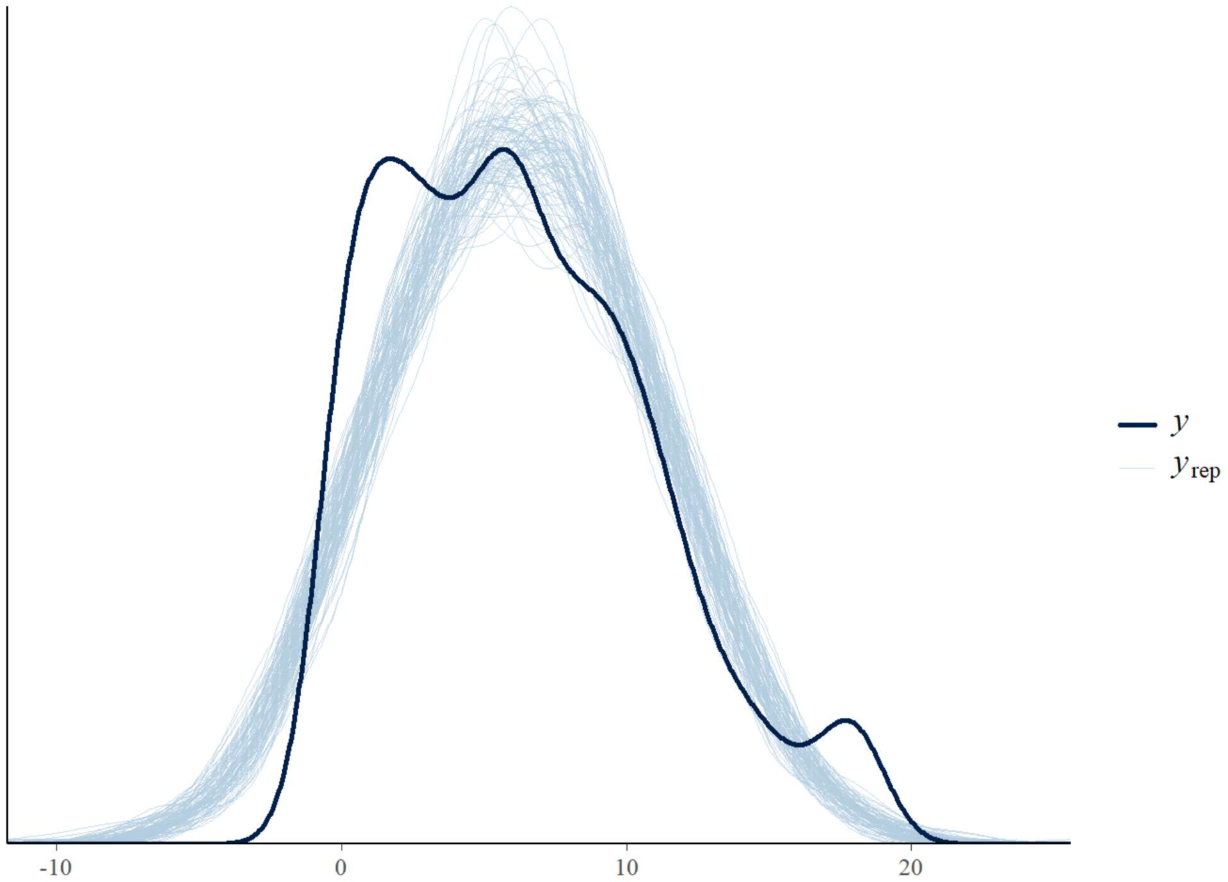

outputs for strongly informative imputed data model

```
strong_prior_model <- readRDS("strong_prior_modelk.rds")

# Print the summary of the model
summary(strong_prior_model)
```

```
##  Family: gaussian
##   Links: mu = identity; sigma = identity
## Formula: k6 ~ time_short + AC_slope_short + S_B_E_slope_short + earth + sae_k + (1 | ID)
##    Data: mids_object (Number of observations: 8800)
##   Draws: 20 chains, each with iter = 15000; warmup = 7500; thin = 1;
##          total post-warmup draws = 150000
##
## Multilevel Hyperparameters:
## ~ID (Number of levels: 55)
##               Estimate Est.Error l-95% CI u-95% CI Rhat Bulk_ESS Tail_ESS
## sd(Intercept)     3.09      0.31     2.57     3.77 1.00     6278    11551
##
## Regression Coefficients:
##                   Estimate Est.Error l-95% CI u-95% CI Rhat Bulk_ESS Tail_ESS
## Intercept             5.37      0.44     4.51     6.23 1.01     4094     8664
## time_short            0.11      0.02     0.07     0.14 1.00    36439    66914
## AC_slope_short        0.03      0.02    -0.01     0.08 1.00    57054    87041
## S_B_E_slope_short     0.11      0.06    -0.01     0.22 1.00    38251    69269
## earth                 1.86      0.14     1.59     2.13 1.00    51387    82992
## sae_k                -0.16      0.05    -0.26    -0.07 1.00    31486    60013
##
## Further Distributional Parameters:
##       Estimate Est.Error l-95% CI u-95% CI Rhat Bulk_ESS Tail_ESS
## sigma     3.36      0.03     3.31     3.41 1.00    89508   101389
##
## Draws were sampled using sampling(NUTS). For each parameter, Bulk_ESS
## and Tail_ESS are effective sample size measures, and Rhat is the potential
## scale reduction factor on split chains (at convergence, Rhat = 1).
```

```
# Test if the coefficient of AC_slope is equal to the coefficient of sae_k
hypothesiss1 <- hypothesis(strong_prior_model, "AC_slope_short = sae_k")

# Display the results
print(hypothesiss1)
```

```
## Hypothesis Tests for class b:
##                 Hypothesis Estimate Est.Error CI.Lower CI.Upper Evid.Ratio
## 1 (AC_slope_short)-... = 0     0.19      0.05     0.11     0.28         NA
##   Post.Prob Star
## 1        NA    *
## ---
## 'CI': 90%-CI for one-sided and 95%-CI for two-sided hypotheses.
## '*': For one-sided hypotheses, the posterior probability exceeds 95%;
## for two-sided hypotheses, the value tested against lies outside the 95%-CI.
## Posterior probabilities of point hypotheses assume equal prior probabilities.
```

```
# Test if the coefficient of AC_slope is equal to the coefficient of S_B_E_slope
hypothesiss2 <- hypothesis(strong_prior_model, "AC_slope_short = S_B_E_slope_short")

# Display the results
print(hypothesiss2)
```

```
## Hypothesis Tests for class b:
##                 Hypothesis Estimate Est.Error CI.Lower CI.Upper Evid.Ratio
## 1 (AC_slope_short)-... = 0    -0.07      0.05    -0.18     0.03         NA
##   Post.Prob Star
## 1        NA
## ---
## 'CI': 90%-CI for one-sided and 95%-CI for two-sided hypotheses.
## '*': For one-sided hypotheses, the posterior probability exceeds 95%;
## for two-sided hypotheses, the value tested against lies outside the 95%-CI.
## Posterior probabilities of point hypotheses assume equal prior probabilities.
```

```
# Test if the coefficient of S_A_E to the coefficient of S_B_E_slope
hypothesiss3 <- hypothesis(strong_prior_model, "sae_k = S_B_E_slope_short")

# Display the results
print(hypothesiss3)
```

```
## Hypothesis Tests for class b:
##                 Hypothesis Estimate Est.Error CI.Lower CI.Upper Evid.Ratio
## 1 (sae_k)-(S_B_E_sl... = 0    -0.27      0.04    -0.36    -0.18         NA
##   Post.Prob Star
## 1        NA    *
## ---
## 'CI': 90%-CI for one-sided and 95%-CI for two-sided hypotheses.
## '*': For one-sided hypotheses, the posterior probability exceeds 95%;
## for two-sided hypotheses, the value tested against lies outside the 95%-CI.
## Posterior probabilities of point hypotheses assume equal prior probabilities.
```

```
pp_check(strong_prior_model, ndraws = 100)
```

```
## Warning: Using only the first imputed data set. Please interpret the results
## with caution until a more principled approach has been implemented.
```

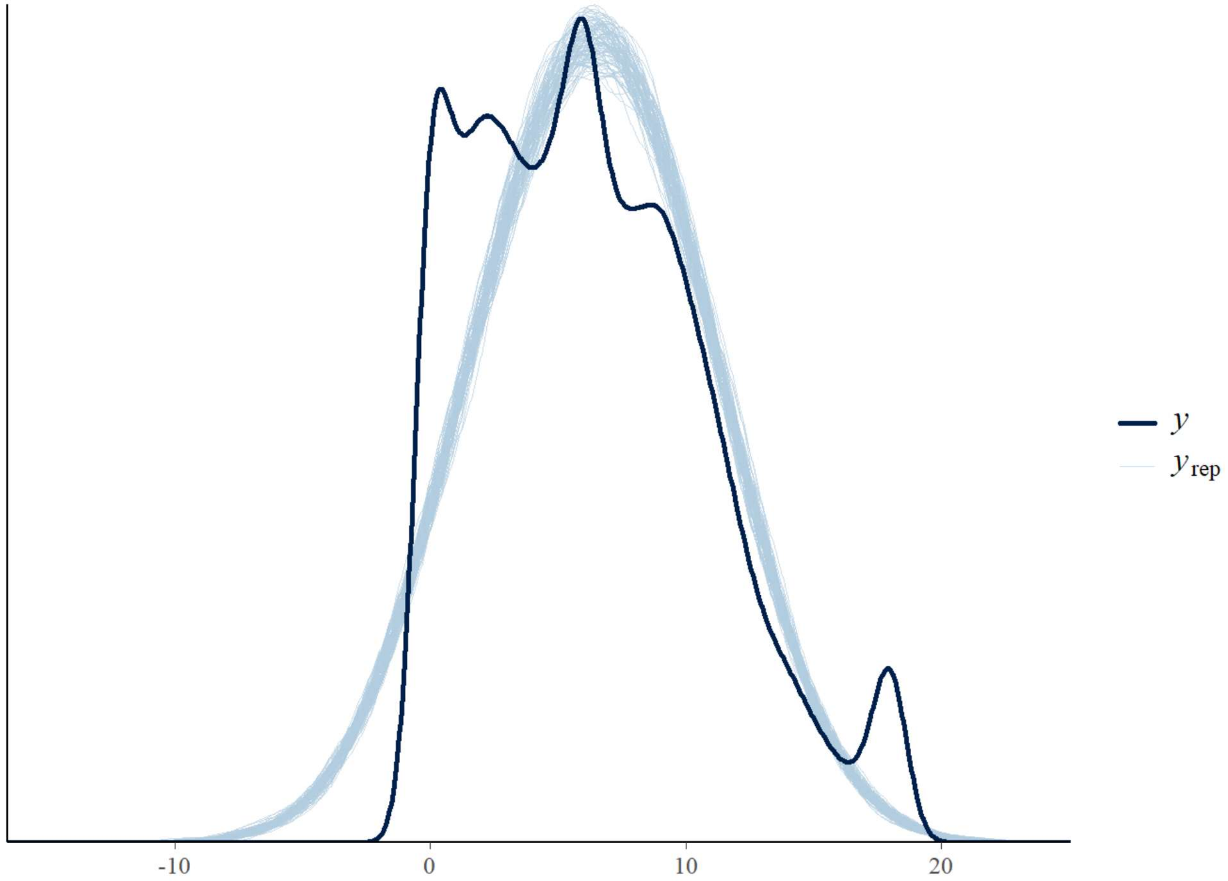

outputs for moderately informative imputed data model

```
moderate_prior_model <- readRDS("moderate_prior_modelk.rds")

# Print the summary of the model
summary(moderate_prior_model)
```

```
##  Family: gaussian
##   Links: mu = identity; sigma = identity
## Formula: k6 ~ time_short + AC_slope_short + S_B_E_slope_short + earth + sae_k + (1 | ID)
##    Data: mids_object (Number of observations: 8800)
##   Draws: 20 chains, each with iter = 15000; warmup = 7500; thin = 1;
##          total post-warmup draws = 150000
##
## Multilevel Hyperparameters:
## ~ID (Number of levels: 55)
##               Estimate Est.Error l-95% CI u-95% CI Rhat Bulk_ESS Tail_ESS
## sd(Intercept)     3.10      0.31     2.58     3.77 1.00     6567    12400
##
## Regression Coefficients:
##                   Estimate Est.Error l-95% CI u-95% CI Rhat Bulk_ESS Tail_ESS
## Intercept             5.35      0.43     4.50     6.20 1.00     4223     8735
## time_short            0.11      0.02     0.08     0.15 1.00    32960    61221
## AC_slope_short        0.03      0.02    -0.02     0.08 1.00    50927    81729
## S_B_E_slope_short     0.09      0.07    -0.04     0.23 1.00    34783    64487
## earth                 1.76      0.19     1.38     2.13 1.00    41113    73602
## sae_k                -0.18      0.06    -0.29    -0.07 1.00    29749    55780
##
## Further Distributional Parameters:
##       Estimate Est.Error l-95% CI u-95% CI Rhat Bulk_ESS Tail_ESS
## sigma     3.36      0.03     3.31     3.41 1.00    90447    99247
##
## Draws were sampled using sampling(NUTS). For each parameter, Bulk_ESS
## and Tail_ESS are effective sample size measures, and Rhat is the potential
## scale reduction factor on split chains (at convergence, Rhat = 1).
```

```
# Test if the coefficient of AC_slope is equal to the coefficient of sae_k
hypothesism1 <- hypothesis(moderate_prior_model, "AC_slope_short = sae_k")

# Display the results
print(hypothesism1)
```

```
## Hypothesis Tests for class b:
##                 Hypothesis Estimate Est.Error CI.Lower CI.Upper Evid.Ratio
## 1 (AC_slope_short)-... = 0     0.21      0.05     0.11     0.31         NA
##   Post.Prob Star
## 1        NA    *
## ---
## 'CI': 90%-CI for one-sided and 95%-CI for two-sided hypotheses.
## '*': For one-sided hypotheses, the posterior probability exceeds 95%;
## for two-sided hypotheses, the value tested against lies outside the 95%-CI.
## Posterior probabilities of point hypotheses assume equal prior probabilities.
```

```
# Test if the coefficient of AC_slope is equal to the coefficient of S_B_E_slope
hypothesism2 <- hypothesis(moderate_prior_model, "AC_slope_short = S_B_E_slope_short")

# Display the results
print(hypothesism2)
```

```
## Hypothesis Tests for class b:
##                  Hypothesis Estimate Est.Error CI.Lower CI.Upper Evid.Ratio
## 1 (AC_slope_short)-... = 0    -0.07      0.06    -0.18     0.05         NA
##   Post.Prob Star
## 1        NA
## ---
## 'CI': 90%-CI for one-sided and 95%-CI for two-sided hypotheses.
## '*': For one-sided hypotheses, the posterior probability exceeds 95%;
## for two-sided hypotheses, the value tested against lies outside the 95%-CI.
## Posterior probabilities of point hypotheses assume equal prior probabilities.
```

```
# Test if the coefficient of S_A_E to the coefficient of S_B_E_slope
hypothesism3 <- hypothesis(moderate_prior_model, "sae_k = S_B_E_slope_short")

# Display the results
print(hypothesism3)
```

```
## Hypothesis Tests for class b:
##                  Hypothesis Estimate Est.Error CI.Lower CI.Upper Evid.Ratio
## 1 (sae_k)-(S_B_E_sl... = 0    -0.27      0.05    -0.36    -0.18         NA
##   Post.Prob Star
## 1        NA    *
## ---
## 'CI': 90%-CI for one-sided and 95%-CI for two-sided hypotheses.
## '*': For one-sided hypotheses, the posterior probability exceeds 95%;
## for two-sided hypotheses, the value tested against lies outside the 95%-CI.
## Posterior probabilities of point hypotheses assume equal prior probabilities.
```

```
pp_check(moderate_prior_model,ndraws = 100)
```

```
## Warning: Using only the first imputed data set. Please interpret the results
## with caution until a more principled approach has been implemented.
```

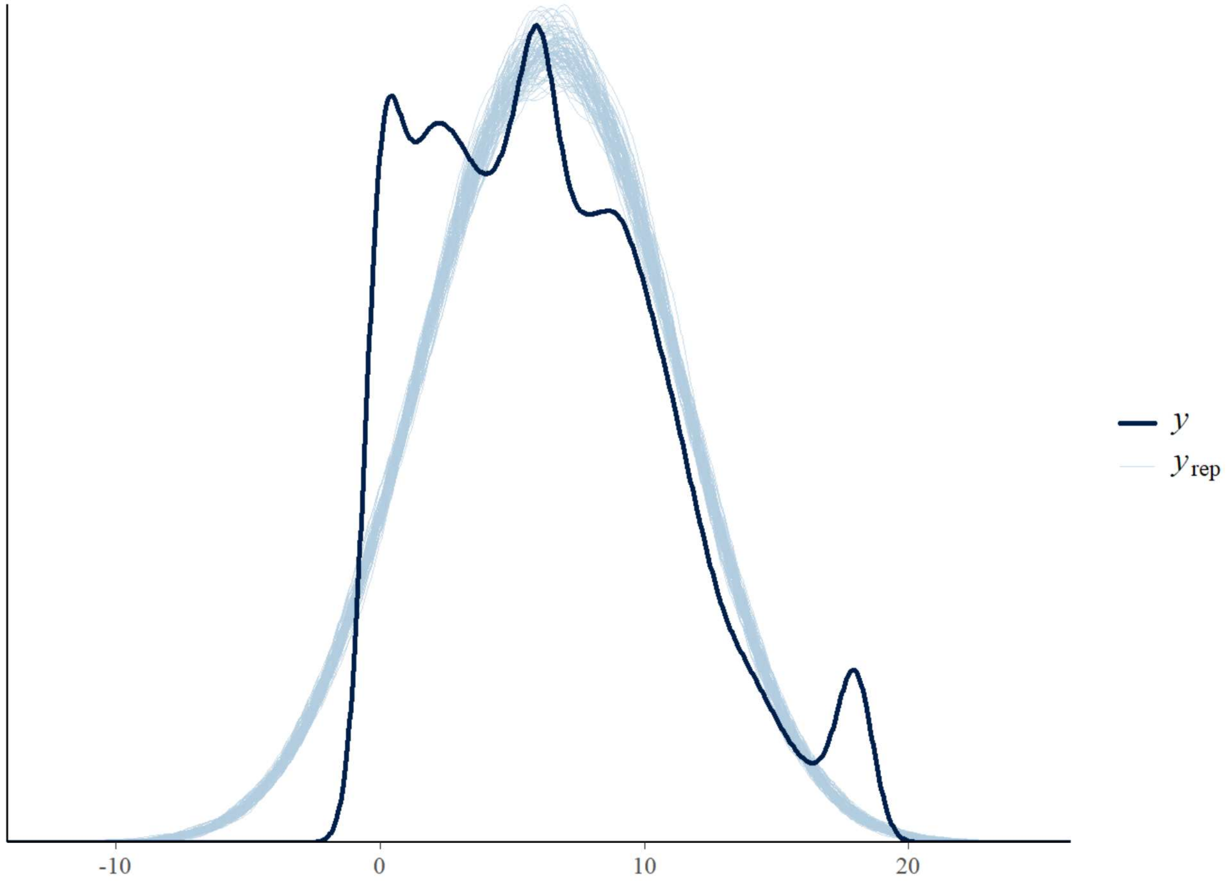

outputs for weakly informative imputed data model

```
weak_prior_model <- readRDS("weak_prior_modelk.rds")

# Print the summary of the model
summary(weak_prior_model)
```

```
##  Family: gaussian
##   Links: mu = identity; sigma = identity
## Formula: k6 ~ time_short + AC_slope_short + S_B_E_slope_short + earth + sae_k + (1 | ID)
##    Data: mids_object (Number of observations: 8800)
##   Draws: 20 chains, each with iter = 15000; warmup = 7500; thin = 1;
##          total post-warmup draws = 150000
##
## Multilevel Hyperparameters:
## ~ID (Number of levels: 55)
##               Estimate Est.Error l-95% CI u-95% CI Rhat Bulk_ESS Tail_ESS
## sd(Intercept)     3.10      0.31     2.57     3.76 1.00     6224    12555
##
## Regression Coefficients:
##                   Estimate Est.Error l-95% CI u-95% CI Rhat Bulk_ESS Tail_ESS
## Intercept             5.35      0.43     4.50     6.21 1.00     3949     8244
## time_short            0.11      0.02     0.08     0.15 1.00    33526    60913
## AC_slope_short        0.03      0.02    -0.02     0.08 1.00    51134    82423
## S_B_E_slope_short     0.09      0.07    -0.04     0.23 1.00    34678    66293
## earth                 1.75      0.20     1.36     2.13 1.00    40402    70644
## sae_k                -0.18      0.06    -0.29    -0.07 1.00    29763    53695
##
## Further Distributional Parameters:
##       Estimate Est.Error l-95% CI u-95% CI Rhat Bulk_ESS Tail_ESS
## sigma     3.36      0.03     3.31     3.41 1.00    88744    97972
##
## Draws were sampled using sampling(NUTS). For each parameter, Bulk_ESS
## and Tail_ESS are effective sample size measures, and Rhat is the potential
## scale reduction factor on split chains (at convergence, Rhat = 1).
```

```
# Test if the coefficient of AC_slope is equal to the coefficient of sae_k
hypothesisw1 <- hypothesis(weak_prior_model, "AC_slope_short = sae_k")

# Display the results
print(hypothesisw1)
```

```
## Hypothesis Tests for class b:
##                 Hypothesis Estimate Est.Error CI.Lower CI.Upper Evid.Ratio
## 1 (AC_slope_short)-... = 0     0.21      0.05     0.11     0.31         NA
##   Post.Prob Star
## 1        NA    *
## ---
## 'CI': 90%-CI for one-sided and 95%-CI for two-sided hypotheses.
## '*': For one-sided hypotheses, the posterior probability exceeds 95%;
## for two-sided hypotheses, the value tested against lies outside the 95%-CI.
## Posterior probabilities of point hypotheses assume equal prior probabilities.
```

```
# Test if the coefficient of AC_slope is equal to the coefficient of S_B_E_slope
hypothesisw2 <- hypothesis(weak_prior_model, "AC_slope_short = S_B_E_slope_short")

# Display the results
print(hypothesisw2)
```

```
## Hypothesis Tests for class b:
##                 Hypothesis Estimate Est.Error CI.Lower CI.Upper Evid.Ratio
## 1 (AC_slope_short)-... = 0    -0.06      0.06    -0.18     0.05         NA
##   Post.Prob Star
## 1        NA
## ---
## 'CI': 90%-CI for one-sided and 95%-CI for two-sided hypotheses.
## '*': For one-sided hypotheses, the posterior probability exceeds 95%;
## for two-sided hypotheses, the value tested against lies outside the 95%-CI.
## Posterior probabilities of point hypotheses assume equal prior probabilities.
```

```
# Test if the coefficient of S_A_E to the coefficient of S_B_E_slope
hypothesisw3 <- hypothesis(weak_prior_model, "sae_k = S_B_E_slope_short")

# Display the results
print(hypothesisw3)
```

```
## Hypothesis Tests for class b:
##                 Hypothesis Estimate Est.Error CI.Lower CI.Upper Evid.Ratio
## 1 (sae_k)-(S_B_E_sl... = 0    -0.27      0.05    -0.36    -0.18         NA
##   Post.Prob Star
## 1        NA    *
## ---
## 'CI': 90%-CI for one-sided and 95%-CI for two-sided hypotheses.
## '*': For one-sided hypotheses, the posterior probability exceeds 95%;
## for two-sided hypotheses, the value tested against lies outside the 95%-CI.
## Posterior probabilities of point hypotheses assume equal prior probabilities.
```

```
pp_check(weak_prior_model, ndraws = 100)
```

```
## Warning: Using only the first imputed data set. Please interpret the results
## with caution until a more principled approach has been implemented.
```

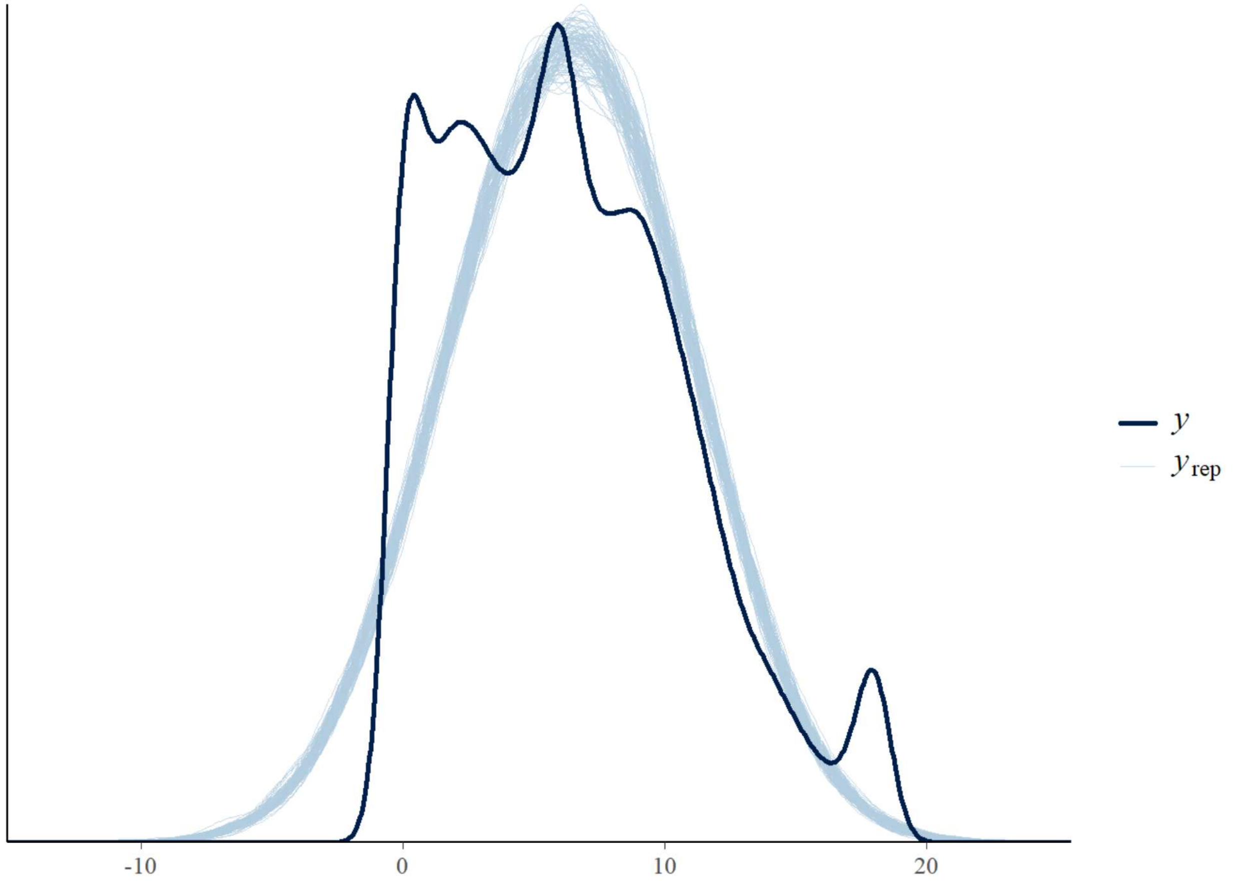


```
# Plot the model diagnostics
plot(moderate_prior_model)
```

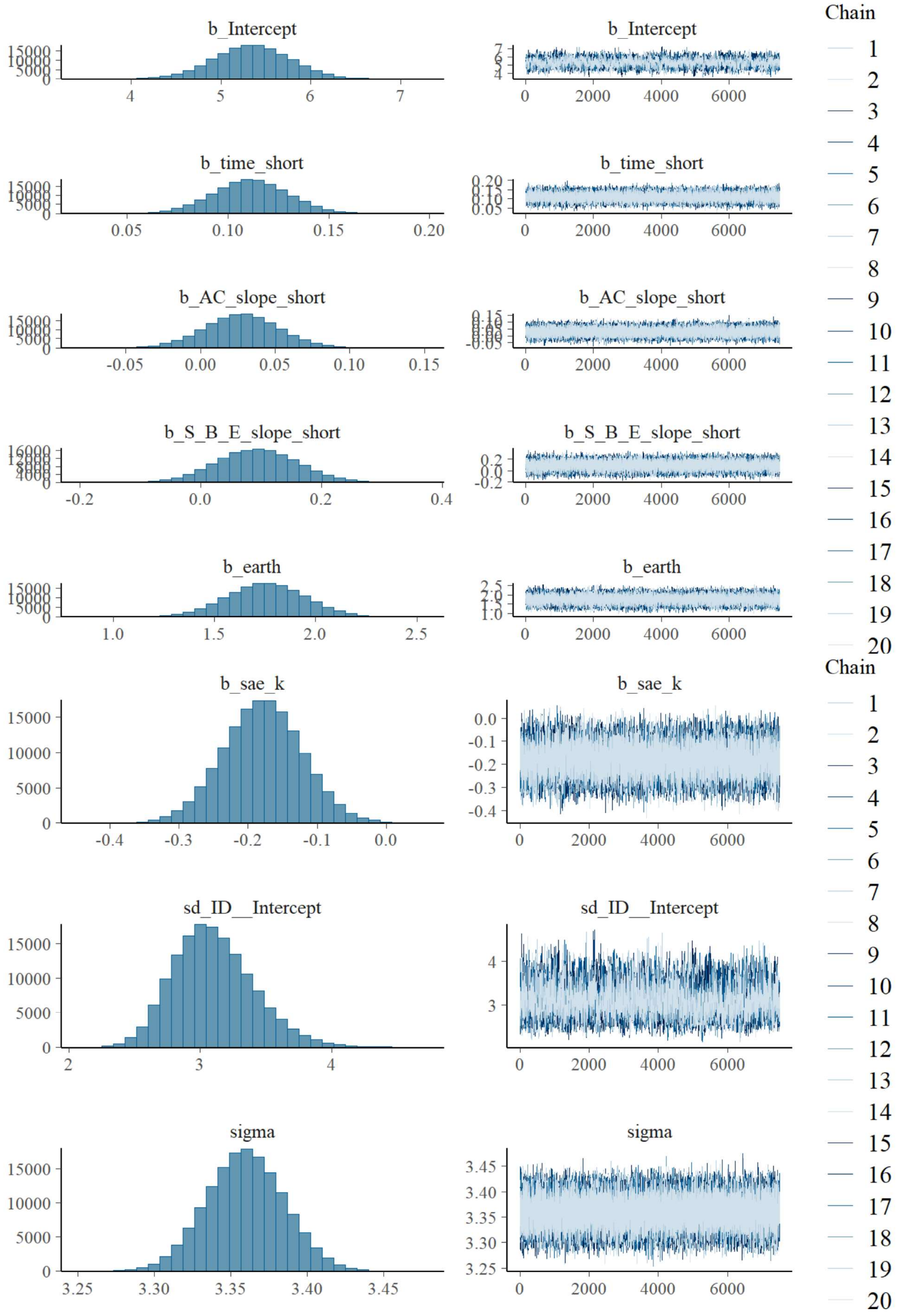


```
# Plot the model diagnostics
plot(model_earth_brms_noT)
```

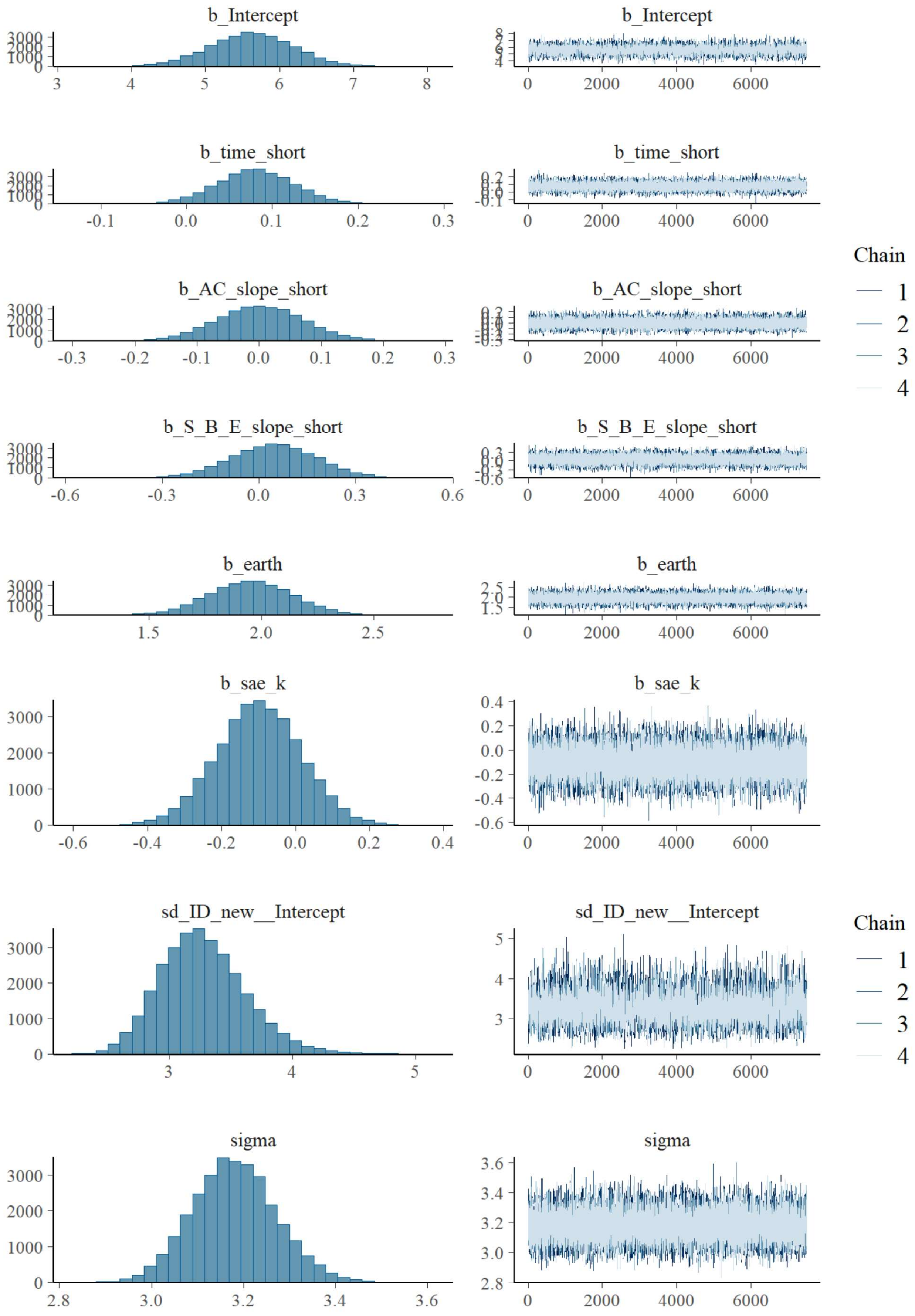


```r
# Plot the model diagnostics
plot(strong_prior_model)
```

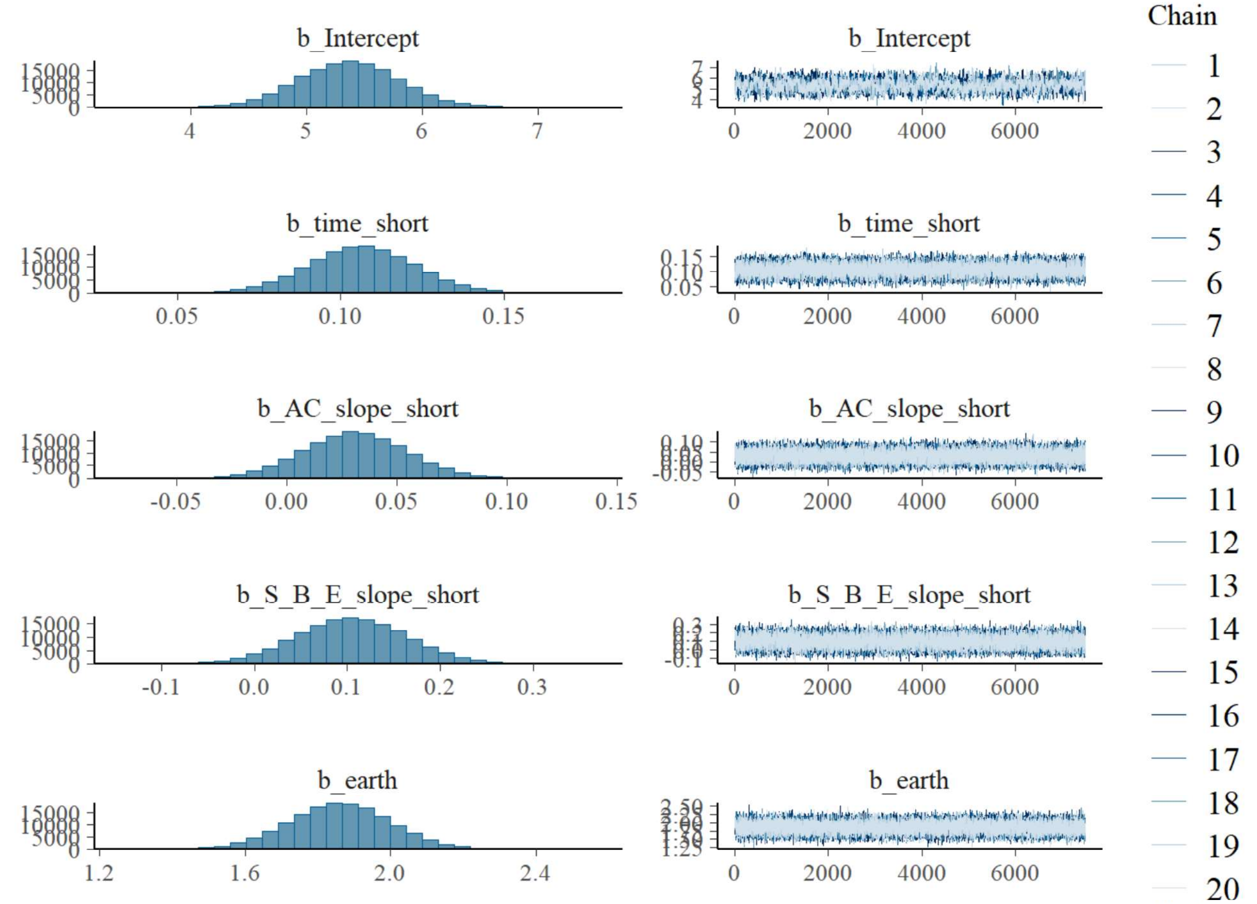

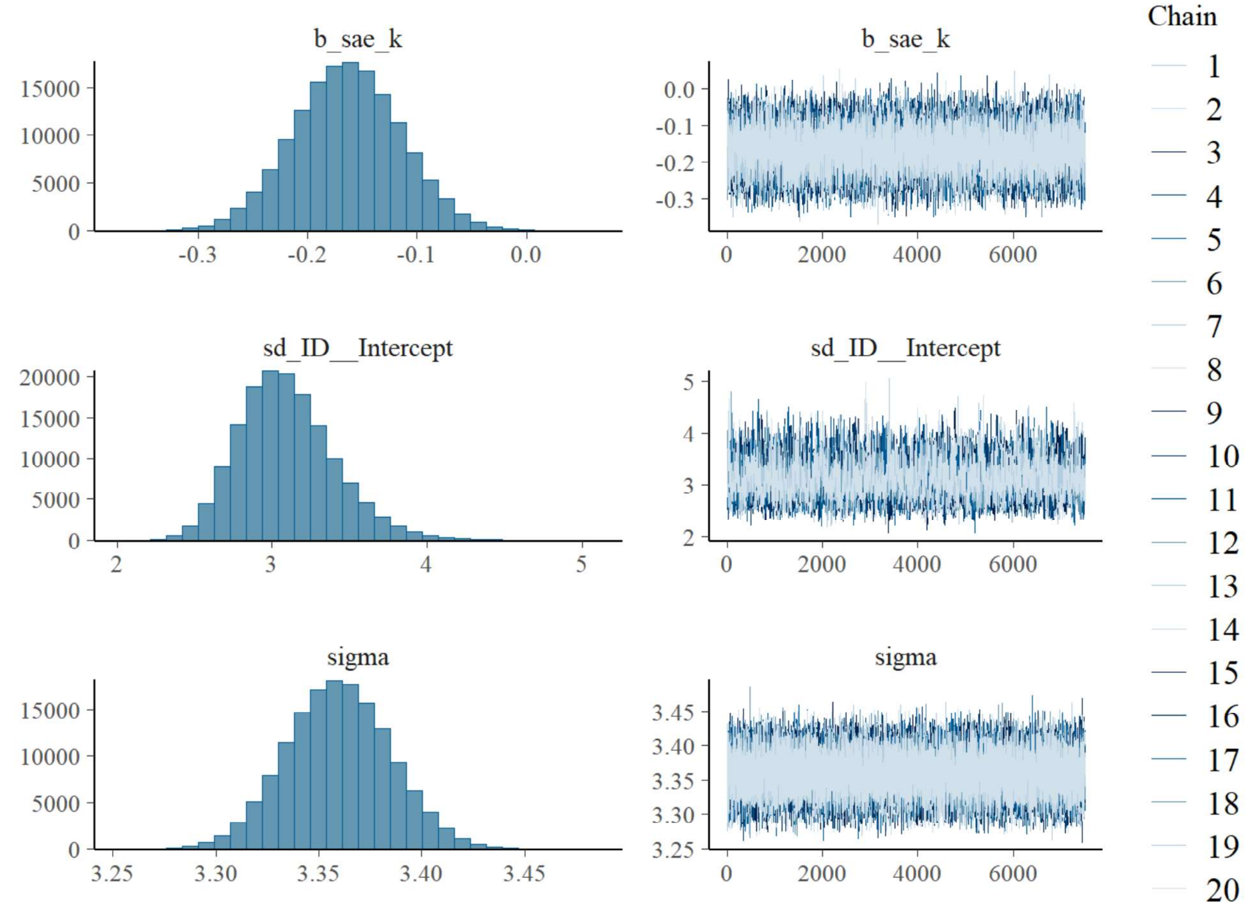


```
# Plot the model diagnostics
plot(weak_prior_model)
```

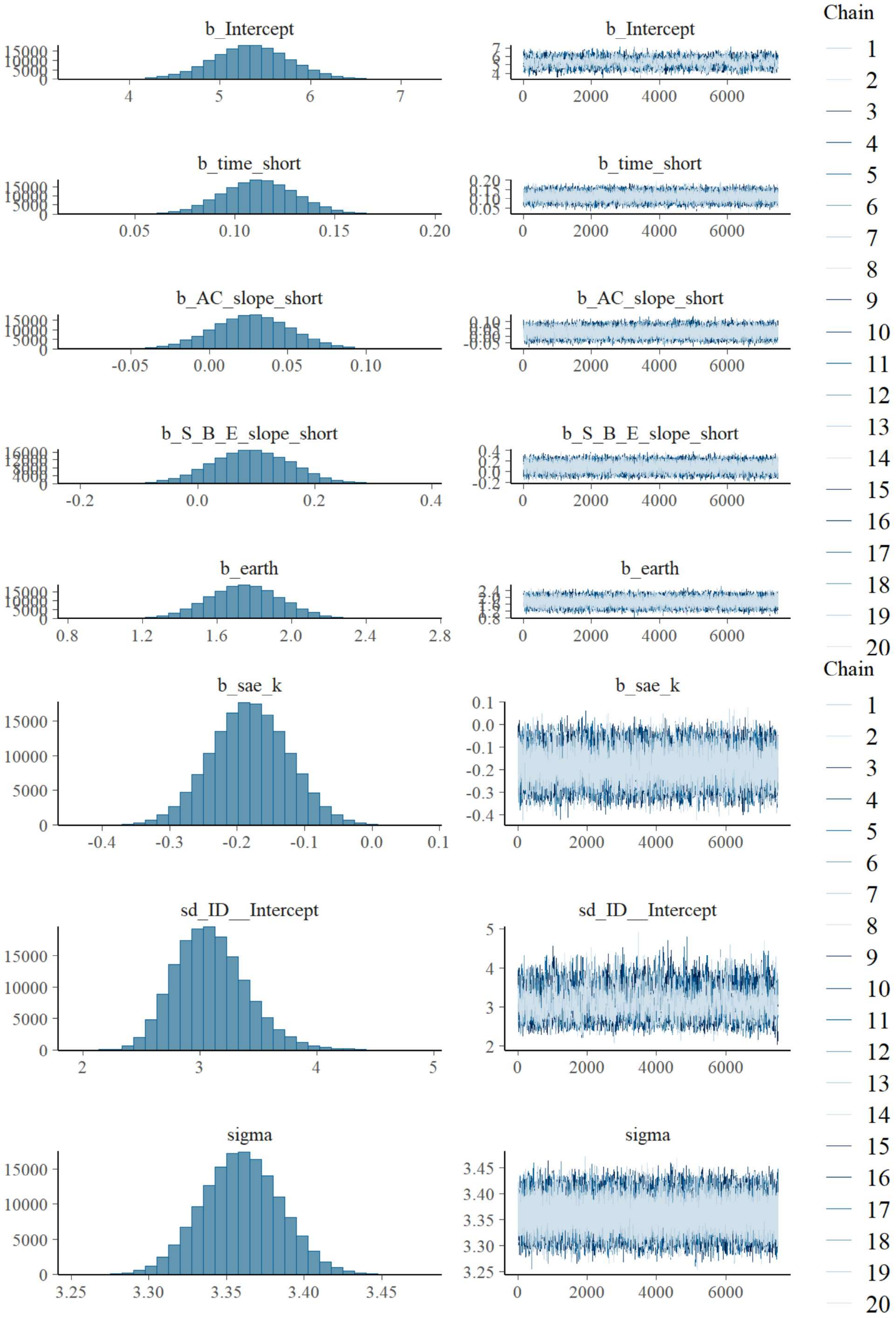
b_Intercept
b_time_short
b_AC_slope_short
b_S_B_E_slope_short
b_earth
b_sae_k
sd_ID__Intercept
sigma
Chain
1
2
3
4
5
6
7
8
9
10
11
12
13
14
15
16
17
18
19
20

Supplementary Materials

Code

Hide

```
library(haven)
CURRENT_data <- read_sav("C:/Users/FINAL TURKIYE-SYRIA PRACTITIONER DATA/CURRENT data.sav")
View(CURRENT_data)
```

Creating the variables for each piecewise slope

Hide

```
library(dplyr)
```

```
Attaching package: ‘dplyr’

The following objects are masked from ‘package:stats’:

    filter, lag

The following objects are masked from ‘package:base’:

    intersect, setdiff, setequal, union
```

Hide

```
# Load the provided table into R
provided_table <- data.frame(
time_short = c(1,2,3,4,5,6,7,8,9,10,13,14,15,16,17,18,NA,NA,NA,NA,NA,NA,NA,NA),
 time_r = c(1,2,3,4,5,6,7,8,9,10,13,14,15,16,17,18,19,20,21,22,23,24,25, 26),
  AC_level = c(1,1,1,1,1,1,1,0,0,0,0,0,0,0,0,0,0,0,0,0,0,0,0, 0),
  AC_slope_short = c(0,1,2,3,4,5,6,0,0,0,0,0,0,0,0,0,NA,NA,NA,NA,NA,NA,NA,NA),
  AC_slope = c(0,1,2,3,4,5,6,0,0,0,0,0,0,0,0,0,0,0,0,0,0,0,0,0),
  S_B_E = c(0,0,0,0,0,0,1,1,1,1,0,0,0,0,0,0,0,0,0,0,0,0,0, 0),
S_B_E_slope_short = c(0,0,0,0,0,0,0,1,2,3,0,0,0,0,0,0,NA,NA,NA,NA,NA,NA,NA,NA),
S_B_E_slope = c(0,0,0,0,0,0,0,1,2,3,0,0,0,0,0,0,0,0,0,0,0,0,0,0),
  S_A_E = c(0,0,0,0,0,0,0,0,0,0,1,1,1,1,1,1,NA,NA,NA,NA,NA,NA,NA,NA),
S_A_E_slope = c(0,0,0,0,0,0,0,0,0,0,1,2,3,4,5,6,NA,NA,NA,NA,NA,NA,NA,NA),
  All_sup = c(0,0,0,0,0,0,1,1,1,1,1,1,1,1,1,1,1,1,1,1,1,1,1, 1),
All_sup_slope = c(0,0,0,0,0,0,0,1,2,3,4,5,6,7,8,9,10,11,12,13,14,15,16,17),
  all_sup_A_E = c(0,0,0,0,0,0,0,0,0,1,1,1,1,1,1,1,1,1,1,1,1,1,1, 1),
all_sup_A_E_slope = c(0,0,0,0,0,0,0,0,0,0,1,2,3,4,5,6,7,8,9,10,11,12,13,14),
  sup_A_E_no_10 = c(0,0,0,0,0,0,0,0,0,0,1,1,1,1,1,1,1,1,1,1,1,1,1, 1),
  sup_A_E_no_10_slope = c(0,0,0,0,0,0,0,0,0,0,0,1,2,3,4,5,6,7,8,9,10,11,12,13),
earth=c(0,0,0,0,0,0,0,0,0,0,1,0,0,0,0,0,NA,NA,NA,NA,NA,NA,NA,NA),
sae_k=c(0,0,0,0,0,0,0,0,0,0,0,1, 2,3,4,5, NA,NA,NA,NA,NA,NA,NA,NA)
)


CURRENT_data <- CURRENT_data %>%
  mutate(across(where(is.numeric), ~na_if(., -999)))



# Merge the provided table with each current data based on time_r and save the modified datas
ets
data_pw <- CURRENT_data %>%
  left_join(provided_table, by = "time_r")

View(data_pw)
```

Run the model with observed data for the outcome variable Compassion Satisfaction

Hide

```
data_pw$ID_new <- as.factor(data_pw$ID_new)

# Load the brms package
library(brms)

priors <- c(
  set_prior("normal(0, 0.2)", class = "b", coef = "time_short"),
  set_prior("normal(0, 0.2)", class = "b", coef = "AC_slope_short"),
  set_prior("normal(-0.1, 0.2)", class = "b", coef = "S_B_E_slope_short"),
  set_prior("normal(0.3, 0.2)", class = "b", coef = "S_A_E_slope"),
  set_prior("normal(37.2, 6.5)", class = "Intercept")
)


# Define the model using brms
model_earth_brms_noT <- brm(c_winsor ~ time_short + AC_slope_short + S_B_E_slope_short + S_A_
E_slope
                            + (1 | ID_new),
                        data = data_pw,
                        family = gaussian(),
                         prior = priors,
                        chains = 4,
                        cores = 4,
                        iter = 8000)
```

```
Warning: Rows containing NAs were excluded from the model.
Compiling Stan program...
Start sampling
```

Hide

```
# Print the summary of the model
summary(model_earth_brms_noT)
```

```
 Family: gaussian
  Links: mu = identity; sigma = identity
Formula: c_winsor ~ time_short + AC_slope_short + S_B_E_slope_short + S_A_E_slope + (1 | ID_n
ew)
   Data: data_pw (Number of observations: 665)
  Draws: 4 chains, each with iter = 8000; warmup = 4000; thin = 1;
         total post-warmup draws = 16000

Multilevel Hyperparameters:
~ID_new (Number of levels: 55)
              Estimate Est.Error l-95% CI u-95% CI Rhat Bulk_ESS Tail_ESS
sd(Intercept)     4.19      0.44     3.43     5.15 1.00     1732     3852

Regression Coefficients:
                  Estimate Est.Error l-95% CI u-95% CI Rhat Bulk_ESS Tail_ESS
Intercept            38.93      0.68    37.60    40.26 1.00     1336     3230
time_short           -0.21      0.04    -0.30    -0.13 1.00     7622    10298
AC_slope_short       -0.07      0.07    -0.20     0.07 1.00    12856    12515
S_B_E_slope_short    -0.11      0.13    -0.37     0.16 1.00    10949    12127
S_A_E_slope           0.27      0.11     0.05     0.49 1.00     7718    10131

Further Distributional Parameters:
      Estimate Est.Error l-95% CI u-95% CI Rhat Bulk_ESS Tail_ESS
sigma     3.20      0.09     3.03     3.39 1.00    14217    12020

Draws were sampled using sampling(NUTS). For each parameter, Bulk_ESS
and Tail_ESS are effective sample size measures, and Rhat is the potential
scale reduction factor on split chains (at convergence, Rhat = 1).
```

Hide

```
# Test if the coefficient of AC_slope is equal to the coefficient of S_A_E_slope
hypothesis3 <- hypothesis(model_earth_brms_noT, "AC_slope_short = S_A_E_slope")

# Display the results
print(hypothesis3)
```

```
Hypothesis Tests for class b:
```

Hypothesis	Estimate	Est.Error	CI.Lower	CI.Upper	Evid.Ratio	Post.Prob
<chr>	<dbl>	<dbl>	<dbl>	<dbl>	<dbl>	<dbl>
(AC_slope_short)-... = 0	-0.34	0.12	-0.58	-0.09	*NA*	*NA*

1 row

```
---
'CI': 90%-CI for one-sided and 95%-CI for two-sided hypotheses.
'*': For one-sided hypotheses, the posterior probability exceeds 95%;
for two-sided hypotheses, the value tested against lies outside the 95%-CI.
Posterior probabilities of point hypotheses assume equal prior probabilities.
```

Hide

```
# Test if the coefficient of AC_slope is equal to the coefficient of S_B_E_slope
hypothesis4 <- hypothesis(model_earth_brms_noT, "AC_slope_short = S_B_E_slope_short")

# Display the results
print(hypothesis4)
```

```
Hypothesis Tests for class b:
```

Hypothesis <chr>	Estimate <dbl>	Est.Error <dbl>	CI.Lower <dbl>	CI.Upper <dbl>	Evid.Ratio <dbl>	Post.Prob <dbl>
(AC_slope_short)-... = 0	0.04	0.13	-0.21	0.3	NA	NA

1 row

```
---
'CI': 90%-CI for one-sided and 95%-CI for two-sided hypotheses.
'*': For one-sided hypotheses, the posterior probability exceeds 95%;
for two-sided hypotheses, the value tested against lies outside the 95%-CI.
Posterior probabilities of point hypotheses assume equal prior probabilities.
```

Hide

```
# Plot the model diagnostics
plot(model_earth_brms_noT)
```

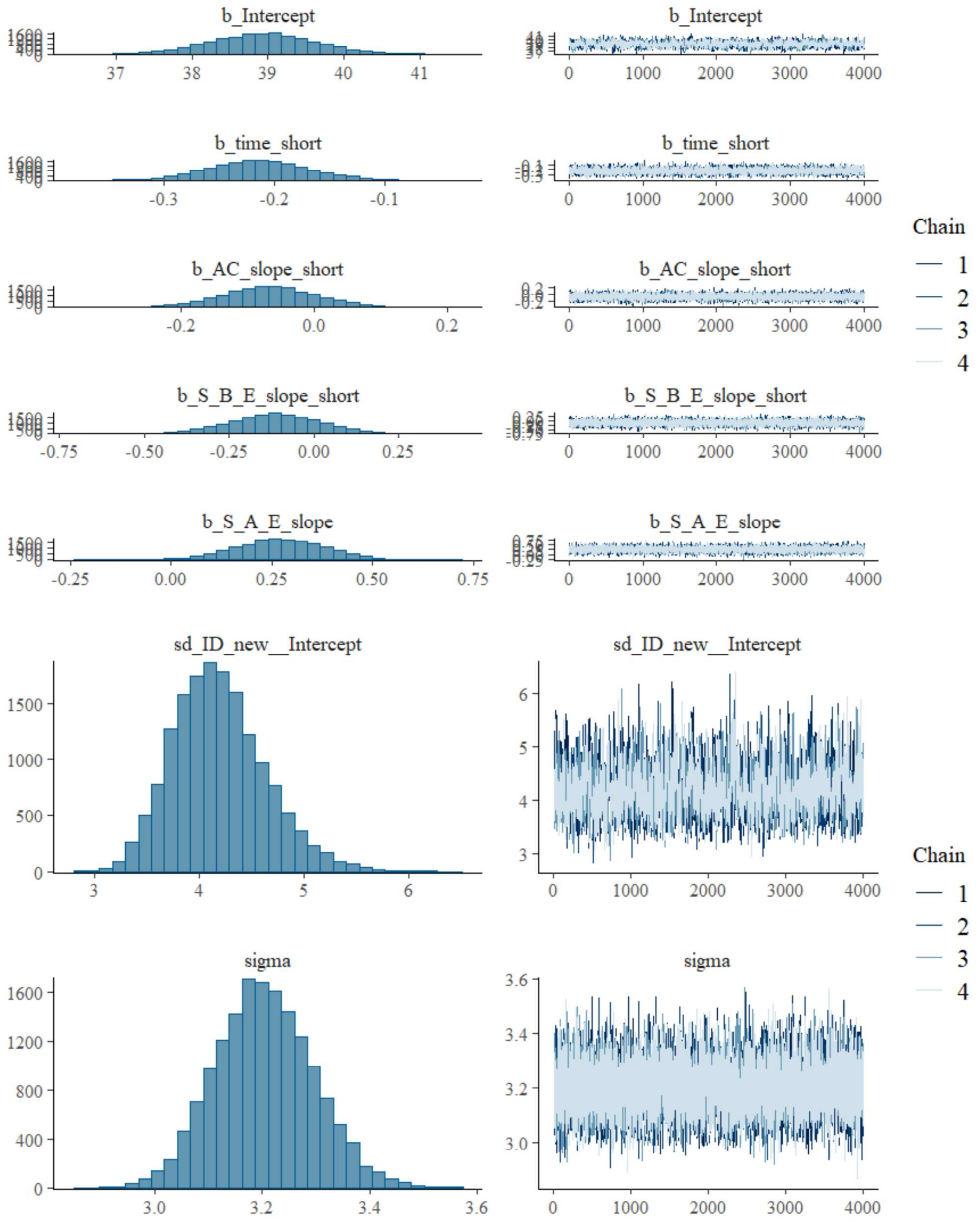

Hide

```
pp_check(model_earth_brms_noT)
```

```
Using 10 posterior draws for ppc type 'dens_overlay' by default.
```

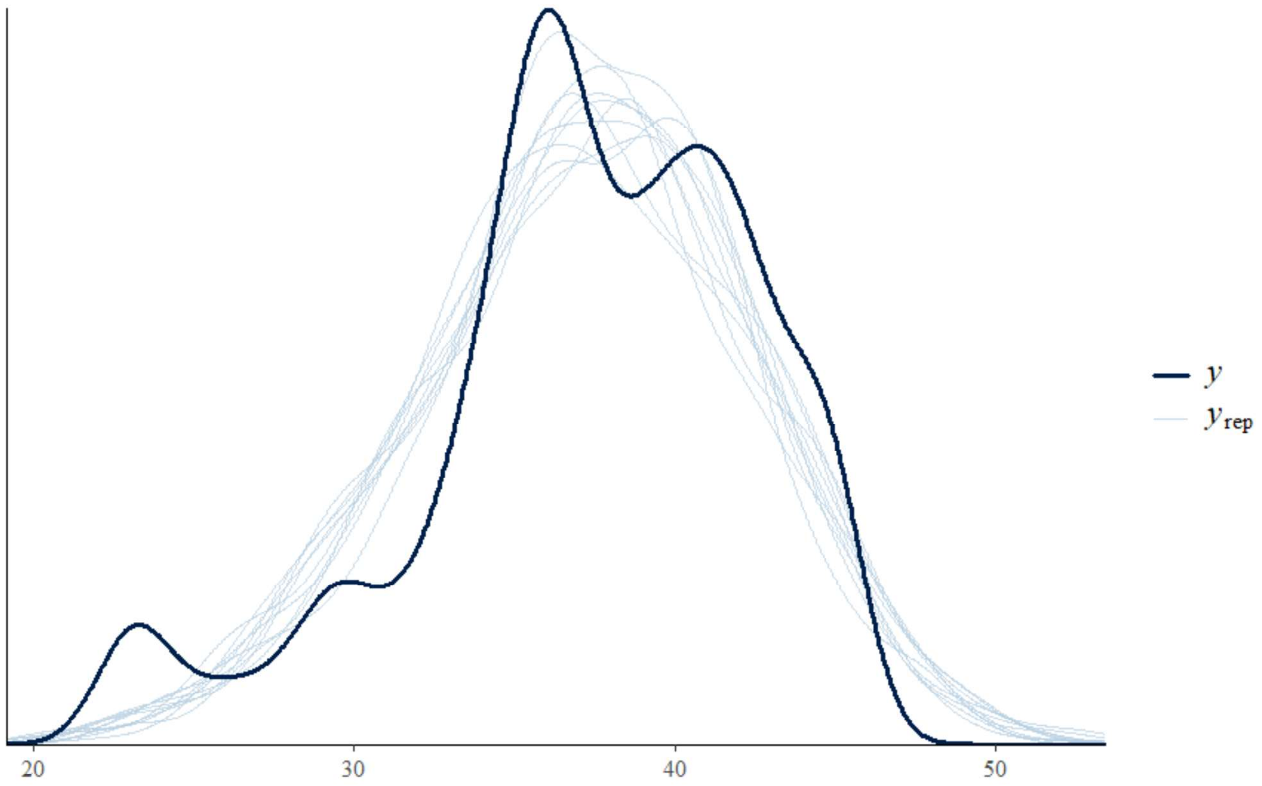

Hide

```
NA
```

BRING IN THE IMPUTED data sets from Mplus

Hide

```
library(dplyr)
library(tidyr)
library(purrr)



# Define the variable names
variable_names <- c("ID", "org", "age", "gend", "trauma", "c_win1", "c_win2", "c_win3", "c_wi
n4", "c_win5", "c_win6", "c_win7", "c_win8", "c_win9", "c_win10",
"c_win13", "c_win14", "c_win15", "c_win16", "c_win17", "c_win18", "c_win19", "c_win20", "c_wi
n21", "c_win22",
"c_win23", "c_win24", "c_win25", "c_win26", "k61", "k62", "k63", "k64", "k65", "k66",
"k67", "k68", "k69", "k610", "k613", "k614", "k615", "k616", "k617", "k618",
```

```
"k619", "k620", "k621", "k622", "k623", "k624", "k625", "k626")

# Assign column names to each data frame
colnames(impute1) <- variable_names
colnames(impute2) <- variable_names
colnames(impute3) <- variable_names
colnames(impute4) <- variable_names
colnames(impute5) <- variable_names
colnames(impute6) <- variable_names
colnames(impute7) <- variable_names
colnames(impute8) <- variable_names
colnames(impute9) <- variable_names
colnames(impute10) <- variable_names


# Verify the column names have been added correctly
head(impute1)
head(impute2)
head(impute3)
head(impute4)
head(impute5)

# Add imputation identifiers
impute1$.imp <- 1
impute2$.imp <- 2
impute3$.imp <- 3
impute4$.imp <- 4
impute5$.imp <- 5
impute6$.imp <- 6
impute7$.imp <- 7
impute8$.imp <- 8
impute9$.imp <- 9
impute10$.imp <- 10

# Combine the datasets into a single data frame
combined_data <- bind_rows(impute1, impute2, impute3, impute4, impute5, impute6,impute7, impu
te8, impute9, impute10)

View(combined_data)
# Load necessary libraries
library(dplyr)
library(tidyr)

# Assuming combined_data is your original data frame
# Pivot the data from wide to long format for c_win and k6 variables
long_data <- combined_data %>%
  pivot_longer(
    cols = starts_with("c_win"),  # Select columns that start with "c_win"
    names_to = "time_r",          # Name of the new long format variable
    values_to = "c_winsor"        # Name of the values in the new long format
  ) %>%
  # Create a numeric time_r variable from the column names
  mutate(time_r = as.numeric(gsub("c_win", "", time_r))) %>%
  pivot_longer(
    cols = starts_with("k6"),     # Select columns that start with "k6"
    names_to = "time_r_k6",       # Temporary name to avoid conflict
```

```
    values_to = "k6"              # Name of the values in the new long format
  ) %>%
  # Ensure time_r_k6 aligns with time_r
  mutate(time_r_k6 = as.numeric(gsub("k6", "", time_r_k6))) %>%
  filter(time_r == time_r_k6) %>%  # Keep rows where time variables match
  select(-time_r_k6)               # Remove the temporary time_r_k6 variable

# View the resulting long_data
head(long_data)
library(mice)



# Merge with provided_table based on time_r
long_data <- long_data %>%
  left_join(provided_table, by = "time_r")

# Ensure the combined dataset has the original data included
# Use the complete function to include original data in long format
long_data_with_orig <- complete(mice(long_data, maxit = 0), action = "long", include = TRUE)

# Add a unique identifier to each row to avoid duplicate row names
long_data_with_orig <- long_data_with_orig %>%
  mutate(.id = paste0(.imp, "_", row_number()))

# Convert the long format data with original data into a mids object
mids_object <- as.mids(long_data_with_orig)

# View the first few rows of the long_data
head(mids_object)
```

RUN IT AGAIN with the imputed data comparing prior settings

Weakly informative Priors model - model did not converge even after increasing iterations to 15000

Hide

```
library(rstan)
```

```
Warning: package ‘rstan’ was built under R version 4.4.1
Loading required package: StanHeaders
Warning: package ‘StanHeaders’ was built under R version 4.4.1

rstan version 2.32.6 (Stan version 2.32.2)

For execution on a local, multicore CPU with excess RAM we recommend calling
options(mc.cores = parallel::detectCores()).
To avoid recompilation of unchanged Stan programs, we recommend calling
rstan_options(auto_write = TRUE)
For within-chain threading using `reduce_sum()` or `map_rect()` Stan functions,
change `threads_per_chain` option:
rstan_options(threads_per_chain = 1)

Do not specify '-march=native' in 'LOCAL_CPPFLAGS' or a Makevars file

Attaching package: ‘rstan’

The following object is masked from ‘package:tidyr’:

    extract

The following object is masked from ‘package:coda’:

    traceplot

The following objects are masked from ‘package:posterior’:

    ess_bulk, ess_tail
```

Hide

```
library(brms)
library(parallel)
rstan_options(auto_write = TRUE)
options(mc.cores = 4)


# Define prior settings

  weakly_informative_priors = c(
   set_prior("normal(0, 10)", class = "b", coef = "time_short"),
   set_prior("normal(0, 10)", class = "b", coef = "AC_slope_short"),
  set_prior("normal(0, 10)", class = "b", coef = "S_B_E_slope_short"),
  set_prior("normal(0, 10)", class = "b", coef = "S_A_E_slope"),
   set_prior("normal(37.2, 20)", class = "Intercept"))

# Fit models for weak prior setting

  weak_prior_model <- brm_multiple(
    formula = c_winsor ~ time_short + AC_slope_short + S_B_E_slope_short + S_A_E_slope +  (1
| ID),
    family = gaussian(),
    data = mids_object,
    prior = weakly_informative_priors ,
    seed = 112,
    chains = 4,
    iter = 15000,
  )
```

```
Compiling the C++ model
Warning: Rows containing NAs were excluded from the model.
Fitting imputed model 1
Warning: Rows containing NAs were excluded from the model.
Start sampling
Fitting imputed model 2
Warning: Rows containing NAs were excluded from the model.
Start sampling
Fitting imputed model 3
Warning: Rows containing NAs were excluded from the model.
Start sampling
Fitting imputed model 4
Warning: Rows containing NAs were excluded from the model.
Start sampling
Fitting imputed model 5
Warning: Rows containing NAs were excluded from the model.
Start sampling
Warning: Bulk Effective Samples Size (ESS) is too low, indicating posterior means and medians
may be unreliable.
Running the chains for more iterations may help. See
https://mc-stan.org/misc/warnings.html#bulk-ess
```

outputs for weakly informative imputed data model

Hide

```
# Print the summary of the model
summary(weak_prior_model)
```

```
 Family: gaussian
  Links: mu = identity; sigma = identity
Formula: c_winsor ~ time_short + AC_slope_short + S_B_E_slope_short + S_A_E_slope + (1 | ID)
   Data: mids_object (Number of observations: 8800)
  Draws: 20 chains, each with iter = 15000; warmup = 7500; thin = 1;
         total post-warmup draws = 150000

Multilevel Hyperparameters:
~ID (Number of levels: 55)
              Estimate Est.Error l-95% CI u-95% CI Rhat Bulk_ESS Tail_ESS
sd(Intercept)     4.18      0.41     3.46     5.09 1.00     4254     7511

Regression Coefficients:
                  Estimate Est.Error l-95% CI u-95% CI Rhat Bulk_ESS Tail_ESS
Intercept            39.06      0.57    37.93    40.19 1.01     2473     4649
time_short           -0.23      0.01    -0.26    -0.20 1.00    25655    50370
AC_slope_short       -0.11      0.02    -0.15    -0.06 1.00    40692    67065
S_B_E_slope_short    -0.11      0.06    -0.22     0.01 1.00    29032    55796
S_A_E_slope           0.26      0.04     0.18     0.35 1.00    23178    45655

Further Distributional Parameters:
      Estimate Est.Error l-95% CI u-95% CI Rhat Bulk_ESS Tail_ESS
sigma     3.35      0.03     3.30     3.40 1.00    52829    76364

Draws were sampled using sampling(NUTS). For each parameter, Bulk_ESS
and Tail_ESS are effective sample size measures, and Rhat is the potential
scale reduction factor on split chains (at convergence, Rhat = 1).
```

Hide

```
# Test if the coefficient of AC_slope is equal to the coefficient of S_A_E_slope
hypothesisw1 <- hypothesis(weak_prior_model, "AC_slope_short = S_A_E_slope")

# Display the results
print(hypothesisw1)
```

```
Hypothesis Tests for class b:
```

Hypothesis <chr>	Estimate <dbl>	Est.Error <dbl>	CI.Lower <dbl>	CI.Upper <dbl>	Evid.Ratio <dbl>	Post.Prob <dbl>
(AC_slope_short)-... = 0	-0.37	0.04	-0.45	-0.28	NA	NA

1 row

```
---
'CI': 90%-CI for one-sided and 95%-CI for two-sided hypotheses.
'*': For one-sided hypotheses, the posterior probability exceeds 95%;
for two-sided hypotheses, the value tested against lies outside the 95%-CI.
Posterior probabilities of point hypotheses assume equal prior probabilities.
```

Hide

```
# Test if the coefficient of AC_slope is equal to the coefficient of S_B_E_slope
hypothesisw2 <- hypothesis(weak_prior_model, "AC_slope_short = S_B_E_slope_short")

# Display the results
print(hypothesisw2)
```

```
Hypothesis Tests for class b:
```

Hypothesis <chr>	Estimate <dbl>	Est.Error <dbl>	CI.Lower <dbl>	CI.Upper <dbl>	Evid.Ratio <dbl>	Post.Prob <dbl>
(AC_slope_short)-... = 0	0	0.05	-0.1	0.1	NA	NA

1 row

```
---
'CI': 90%-CI for one-sided and 95%-CI for two-sided hypotheses.
'*': For one-sided hypotheses, the posterior probability exceeds 95%;
for two-sided hypotheses, the value tested against lies outside the 95%-CI.
Posterior probabilities of point hypotheses assume equal prior probabilities.
```

Hide

```
# Test if the coefficient of S_A_E to the coefficient of S_B_E_slope
hypothesisw3 <- hypothesis(weak_prior_model, "S_A_E_slope = S_B_E_slope_short")

# Display the results
print(hypothesisw3)
```

```
Hypothesis Tests for class b:
```

Hypothesis <chr>	Estimate <dbl>	Est.Error <dbl>	CI.Lower <dbl>	CI.Upper <dbl>	Evid.Ratio <dbl>	Post.Prob <dbl>
(S_A_E_slope)-(S_... = 0	0.37	0.05	0.28	0.46	NA	NA

1 row

```
---
'CI': 90%-CI for one-sided and 95%-CI for two-sided hypotheses.
'*': For one-sided hypotheses, the posterior probability exceeds 95%;
for two-sided hypotheses, the value tested against lies outside the 95%-CI.
Posterior probabilities of point hypotheses assume equal prior probabilities.
```

Hide

```
# Plot the model diagnostics
plot(weak_prior_model)
```

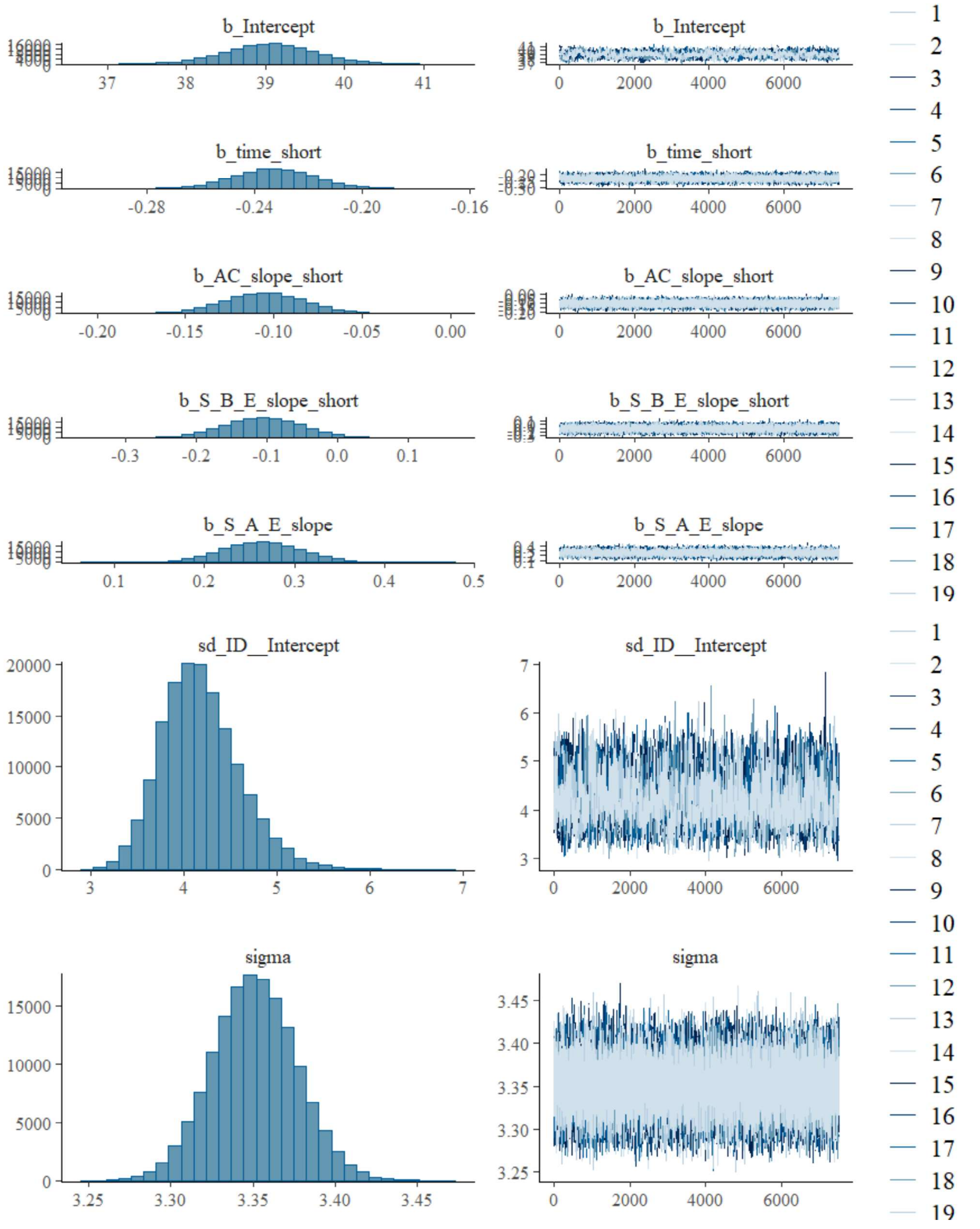

Hide

```
pp_check(weak_prior_model)
```

```
Using 10 posterior draws for ppc type 'dens_overlay' by default.
Warning: Using only the first imputed data set. Please interpret the results with caution unt
il a more principled approach has been implemented.
```

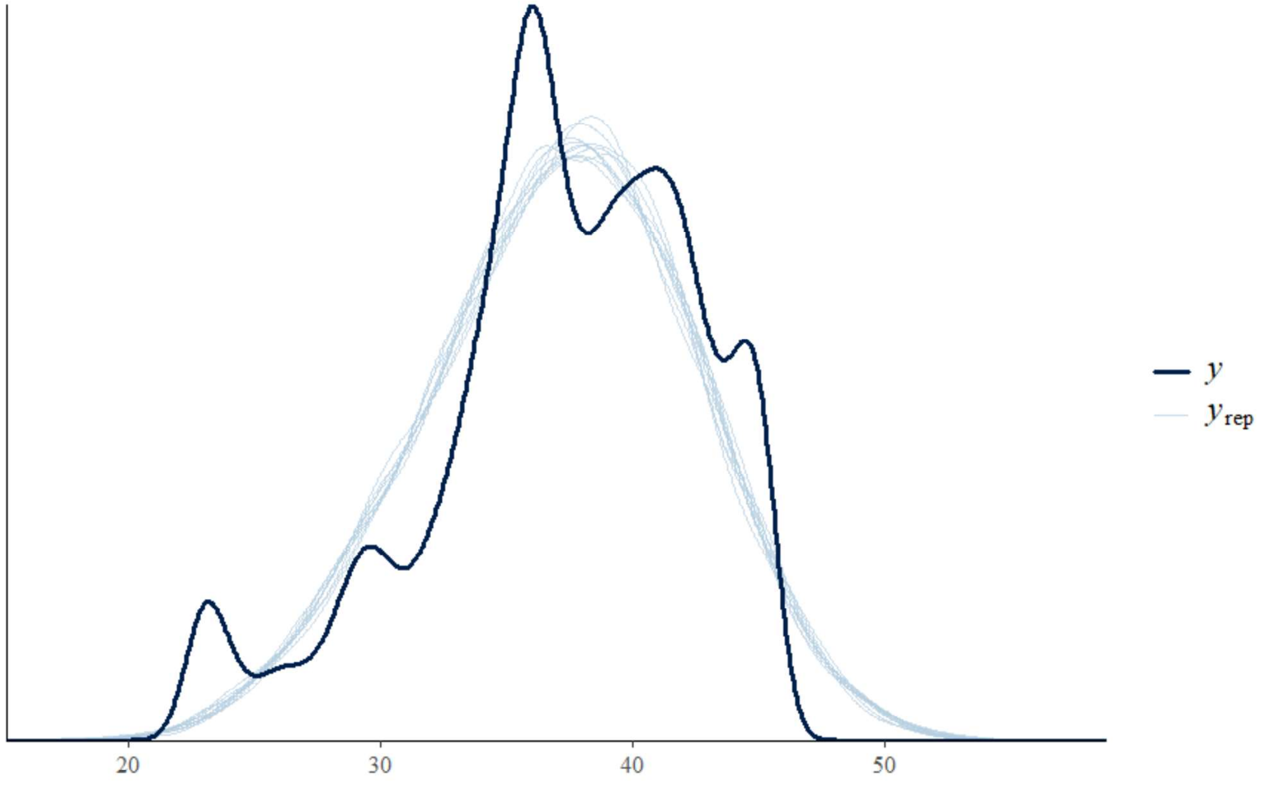

Hide

```
NA
```

Hide

```
# Define prior settings

moderately_informative_priors = c(
    set_prior("normal(0, 1)", class = "b", coef = "time_short"),
    set_prior("normal(0, 1)", class = "b", coef = "AC_slope_short"),
    set_prior("normal(-0.1, 1)", class = "b", coef = "S_B_E_slope_short"),
    set_prior("normal(0.3, 1)", class = "b", coef = "S_A_E_slope"),
    set_prior("normal(37.2, 10)", class = "Intercept"))

# Fit models for weak prior setting

  moderate_prior_model <- brm_multiple(
    formula = c_winsor ~ time_short + AC_slope_short + S_B_E_slope_short + S_A_E_slope +  (1
| ID),
    family = gaussian(),
    data = mids_object,
    prior = moderately_informative_priors ,
    seed = 112,
    chains = 4,
    iter = 15000,
  )
```

```
Compiling the C++ model
Warning: Rows containing NAs were excluded from the model.
Fitting imputed model 1
Warning: Rows containing NAs were excluded from the model.
Start sampling
Fitting imputed model 2
Warning: Rows containing NAs were excluded from the model.
Start sampling
Fitting imputed model 3
Warning: Rows containing NAs were excluded from the model.
Start sampling
Fitting imputed model 4
Warning: Rows containing NAs were excluded from the model.
Start sampling
Fitting imputed model 5
Warning: Rows containing NAs were excluded from the model.
Start sampling
```

outputs for moderately informative imputed data model

Hide

```
# Print the summary of the model
summary(moderate_prior_model)
```

```
 Family: gaussian
  Links: mu = identity; sigma = identity
Formula: c_winsor ~ time_short + AC_slope_short + S_B_E_slope_short + S_A_E_slope + (1 | ID)
   Data: mids_object (Number of observations: 8800)
  Draws: 20 chains, each with iter = 15000; warmup = 7500; thin = 1;
         total post-warmup draws = 150000

Multilevel Hyperparameters:
~ID (Number of levels: 55)
              Estimate Est.Error l-95% CI u-95% CI Rhat Bulk_ESS Tail_ESS
sd(Intercept)     4.17      0.41     3.46     5.07 1.00     4546     8152

Regression Coefficients:
                  Estimate Est.Error l-95% CI u-95% CI Rhat Bulk_ESS Tail_ESS
Intercept            39.06      0.58    37.92    40.19 1.00     2737     5589
time_short           -0.23      0.01    -0.26    -0.20 1.00    27853    52352
AC_slope_short       -0.10      0.02    -0.15    -0.06 1.00    38604    65935
S_B_E_slope_short    -0.11      0.06    -0.22     0.01 1.00    29359    57701
S_A_E_slope           0.26      0.04     0.18     0.35 1.00    24263    46025

Further Distributional Parameters:
      Estimate Est.Error l-95% CI u-95% CI Rhat Bulk_ESS Tail_ESS
sigma     3.35      0.03     3.30     3.40 1.00    51509    75808

Draws were sampled using sampling(NUTS). For each parameter, Bulk_ESS
and Tail_ESS are effective sample size measures, and Rhat is the potential
scale reduction factor on split chains (at convergence, Rhat = 1).
```

Hide

```
# Test if the coefficient of AC_slope is equal to the coefficient of S_A_E_slope
hypothesism1 <- hypothesis(moderate_prior_model, "AC_slope_short = S_A_E_slope")

# Display the results
print(hypothesism1)
```

```
Hypothesis Tests for class b:
```

Hypothesis <chr>	Estimate <dbl>	Est.Error <dbl>	CI.Lower <dbl>	CI.Upper <dbl>	Evid.Ratio <dbl>	Post.Prob <dbl>
(AC_slope_short)-... = 0	-0.37	0.04	-0.45	-0.28	NA	NA

1 row

```
---
'CI': 90%-CI for one-sided and 95%-CI for two-sided hypotheses.
'*': For one-sided hypotheses, the posterior probability exceeds 95%;
for two-sided hypotheses, the value tested against lies outside the 95%-CI.
Posterior probabilities of point hypotheses assume equal prior probabilities.
```

Hide

```
# Test if the coefficient of AC_slope is equal to the coefficient of S_B_E_slope
hypothesism2 <- hypothesis(moderate_prior_model, "AC_slope_short = S_B_E_slope_short")

# Display the results
print(hypothesism2)
```

```
Hypothesis Tests for class b:
```

Hypothesis <chr>	Estimate <dbl>	Est.Error <dbl>	CI.Lower <dbl>	CI.Upper <dbl>	Evid.Ratio <dbl>	Post.Prob <dbl>
(AC_slope_short)-... = 0	0	0.05	-0.1	0.1	NA	NA

1 row

```
---
'CI': 90%-CI for one-sided and 95%-CI for two-sided hypotheses.
'*': For one-sided hypotheses, the posterior probability exceeds 95%;
for two-sided hypotheses, the value tested against lies outside the 95%-CI.
Posterior probabilities of point hypotheses assume equal prior probabilities.
```

Hide

```
# Test if the coefficient of S_A_E to the coefficient of S_B_E_slope
hypothesism3 <- hypothesis(moderate_prior_model, "S_A_E_slope = S_B_E_slope_short")

# Display the results
print(hypothesism3)
```

```
Hypothesis Tests for class b:
```

Hypothesis	Estimate	Est.Error	CI.Lower	CI.Upper	Evid.Ratio	Post.Prob
<chr>	<dbl>	<dbl>	<dbl>	<dbl>	<dbl>	<dbl>
(S_A_E_slope)-(S_... = 0	0.37	0.05	0.28	0.46	*NA*	*NA*

1 row

```
---
'CI': 90%-CI for one-sided and 95%-CI for two-sided hypotheses.
'*': For one-sided hypotheses, the posterior probability exceeds 95%;
for two-sided hypotheses, the value tested against lies outside the 95%-CI.
Posterior probabilities of point hypotheses assume equal prior probabilities.
```

Hide

```
# Plot the model diagnostics
plot(moderate_prior_model)
```

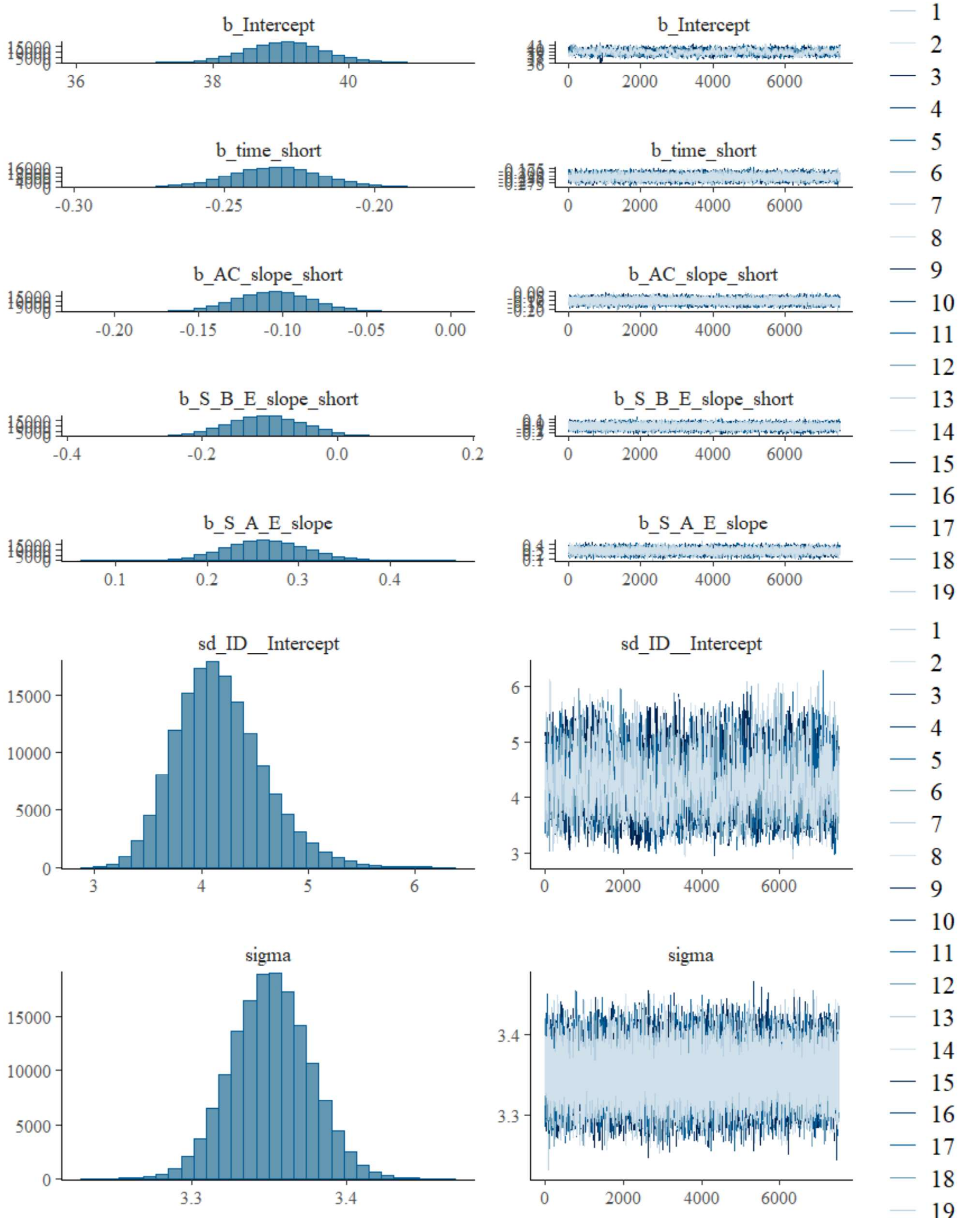

Hide

```
pp_check(moderate_prior_model)
```

```
Using 10 posterior draws for ppc type 'dens_overlay' by default.
Warning: Using only the first imputed data set. Please interpret the results with caution unt
il a more principled approach has been implemented.
```

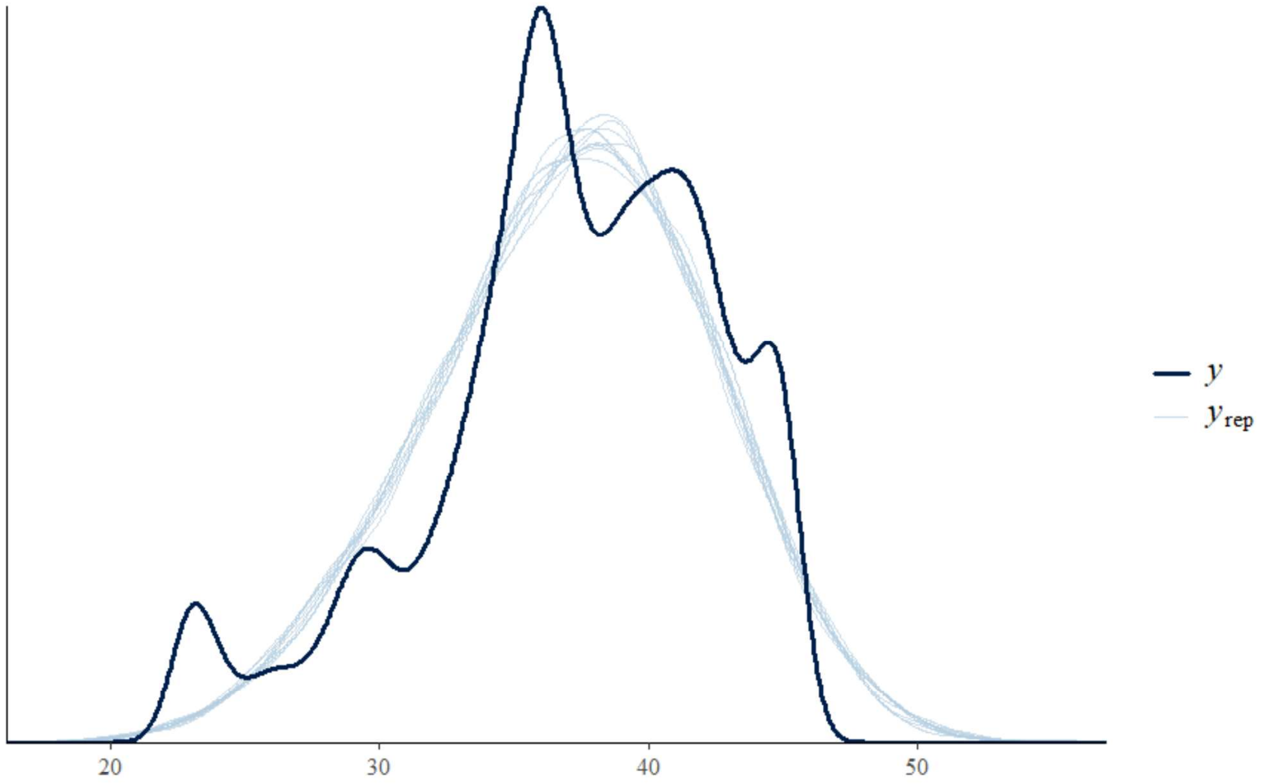

Hide

```
NA
```

Run with Strongly informative priors

Hide

```

# Define prior settings

  strongly_informative_priors = c(
    set_prior("normal(0, 0.2)", class = "b", coef = "time_short"),
    set_prior("normal(0, 0.2)", class = "b", coef = "AC_slope_short"),
    set_prior("normal(-0.1, 0.2)", class = "b", coef = "S_B_E_slope_short"),
    set_prior("normal(0.3, 0.2)", class = "b", coef = "S_A_E_slope"),
    set_prior("normal(37.2, 6.5)", class = "Intercept")  )

# Fit models for moderate prior setting

  strong_prior_model <- brm_multiple(
    formula = c_winsor ~ time_short + AC_slope_short + S_B_E_slope_short + S_A_E_slope +  (1
| ID),
    family = gaussian(),
    data = mids_object,
    prior = strongly_informative_priors ,
    seed = 112,
    chains = 4,
    iter = 15000,
  )
```

outputs for strongly informative imputed data model

Hide

```
# Print the summary of the model
summary(strong_prior_model)
```

```
 Family: gaussian
  Links: mu = identity; sigma = identity
Formula: c_winsor ~ time_short + AC_slope_short + S_B_E_slope_short + S_A_E_slope + (1 | ID)
   Data: mids_object (Number of observations: 8800)
  Draws: 20 chains, each with iter = 15000; warmup = 7500; thin = 1;
         total post-warmup draws = 150000

Multilevel Hyperparameters:
~ID (Number of levels: 55)
              Estimate Est.Error l-95% CI u-95% CI Rhat Bulk_ESS Tail_ESS
sd(Intercept)     4.18      0.42     3.46     5.10 1.00     4090     6921

Regression Coefficients:
                  Estimate Est.Error l-95% CI u-95% CI Rhat Bulk_ESS Tail_ESS
Intercept            39.05      0.58    37.91    40.18 1.01     2395     4679
time_short           -0.23      0.01    -0.26    -0.20 1.00    26790    50714
AC_slope_short       -0.10      0.02    -0.15    -0.06 1.00    40986    68473
S_B_E_slope_short    -0.10      0.05    -0.21     0.00 1.00    28965    56567
S_A_E_slope           0.26      0.04     0.18     0.35 1.00    23149    45907

Further Distributional Parameters:
      Estimate Est.Error l-95% CI u-95% CI Rhat Bulk_ESS Tail_ESS
sigma     3.35      0.03     3.30     3.40 1.00    52516    75331

Draws were sampled using sampling(NUTS). For each parameter, Bulk_ESS
and Tail_ESS are effective sample size measures, and Rhat is the potential
scale reduction factor on split chains (at convergence, Rhat = 1).
```

Hide

```
# Test if the coefficient of AC_slope is equal to the coefficient of S_A_E_slope
hypothesiss1 <- hypothesis(strong_prior_model, "AC_slope_short = S_A_E_slope")

# Display the results
print(hypothesiss1)
```

```
Hypothesis Tests for class b:
```

Hypothesis <chr>	Estimate <dbl>	Est.Error <dbl>	CI.Lower <dbl>	CI.Upper <dbl>	Evid.Ratio <dbl>	Post.Prob <dbl>
(AC_slope_short)-... = 0	-0.37	0.04	-0.45	-0.28	NA	NA

1 row

```
---
'CI': 90%-CI for one-sided and 95%-CI for two-sided hypotheses.
'*': For one-sided hypotheses, the posterior probability exceeds 95%;
for two-sided hypotheses, the value tested against lies outside the 95%-CI.
Posterior probabilities of point hypotheses assume equal prior probabilities.
```

Hide

```
# Test if the coefficient of AC_slope is equal to the coefficient of S_B_E_slope
hypothesiss2 <- hypothesis(strong_prior_model, "AC_slope_short = S_B_E_slope_short")

# Display the results
print(hypothesiss2)
```

```
Hypothesis Tests for class b:
```

Hypothesis <chr>	Estimate <dbl>	Est.Error <dbl>	CI.Lower <dbl>	CI.Upper <dbl>	Evid.Ratio <dbl>	Post.Prob <dbl>
(AC_slope_short)-... = 0	0	0.05	-0.09	0.1	NA	NA

1 row

```
---
'CI': 90%-CI for one-sided and 95%-CI for two-sided hypotheses.
'*': For one-sided hypotheses, the posterior probability exceeds 95%;
for two-sided hypotheses, the value tested against lies outside the 95%-CI.
Posterior probabilities of point hypotheses assume equal prior probabilities.
```

Hide

```
# Test if the coefficient of S_A_E to the coefficient of S_B_E_slope
hypothesiss3 <- hypothesis(strong_prior_model, "S_A_E_slope = S_B_E_slope_short")

# Display the results
print(hypothesiss3)
```

```
Hypothesis Tests for class b:
```

Hypothesis <chr>	Estimate <dbl>	Est.Error <dbl>	CI.Lower <dbl>	CI.Upper <dbl>	Evid.Ratio <dbl>	Post.Prob <dbl>
(S_A_E_slope)-(S_... = 0	0.37	0.04	0.28	0.45	NA	NA

1 row

```
---
'CI': 90%-CI for one-sided and 95%-CI for two-sided hypotheses.
'*': For one-sided hypotheses, the posterior probability exceeds 95%;
for two-sided hypotheses, the value tested against lies outside the 95%-CI.
Posterior probabilities of point hypotheses assume equal prior probabilities.
```

Hide

```
# Plot the model diagnostics
plot(strong_prior_model)
```

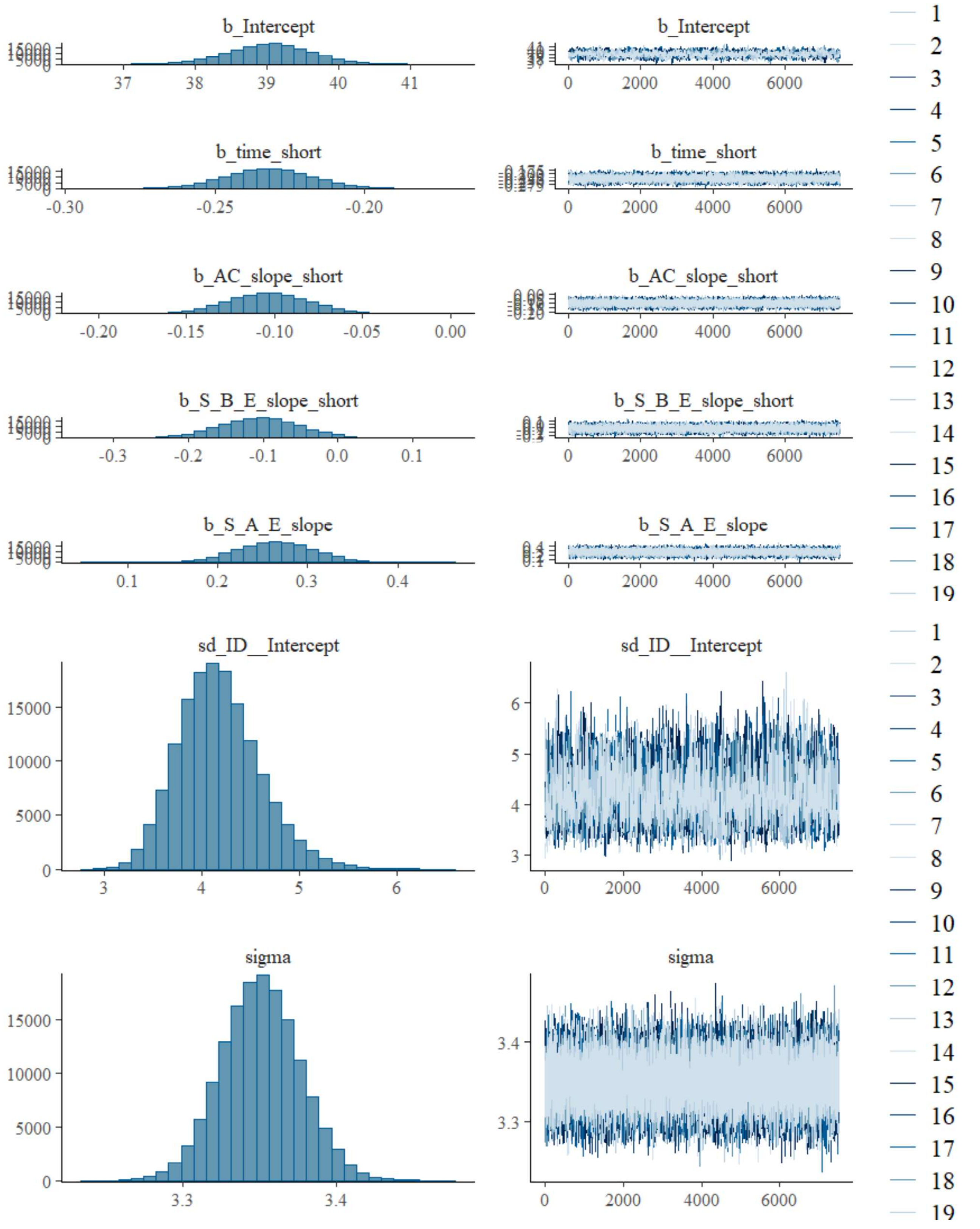

Hide

```
pp_check(strong_prior_model)
```

```
Using 10 posterior draws for ppc type 'dens_overlay' by default.
Warning: Using only the first imputed data set. Please interpret the results with caution unt
il a more principled approach has been implemented.
```

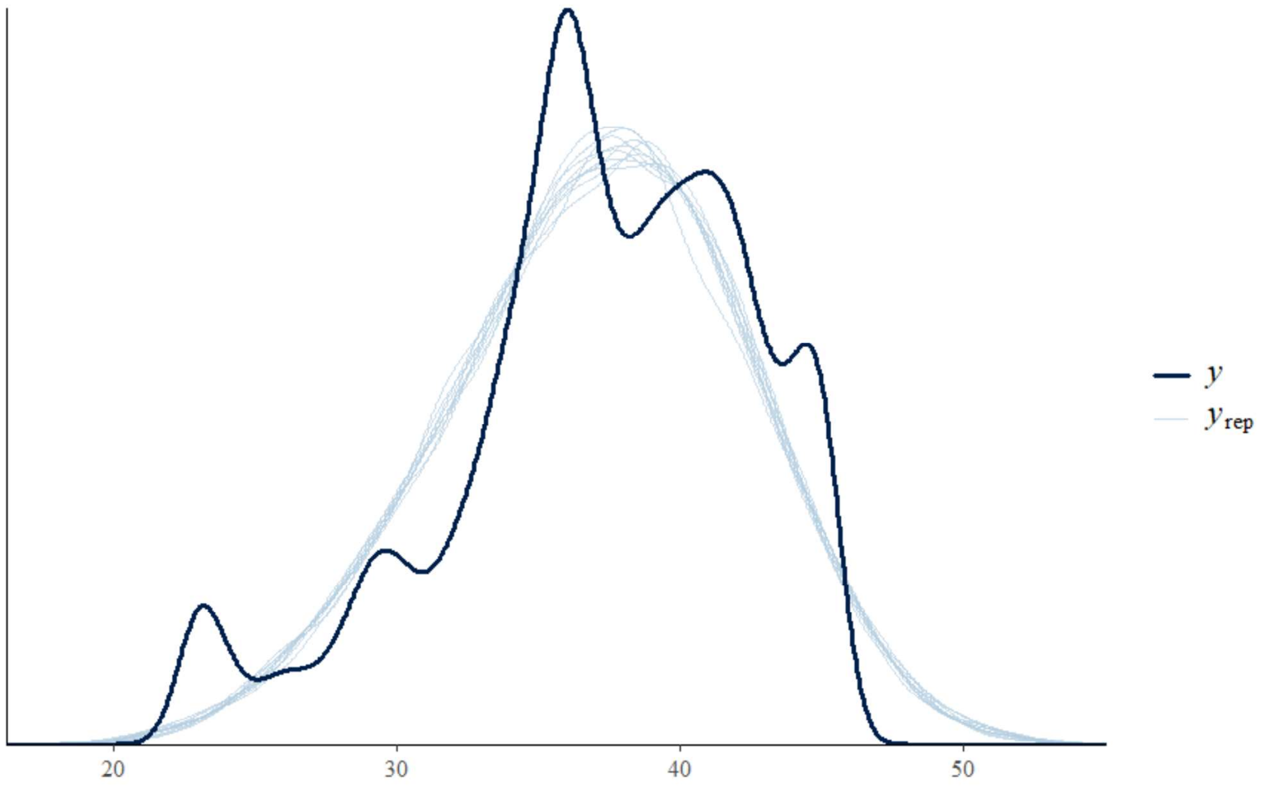

Hide

```
NA
```

LOO

Hide

```
library(brms)
library(loo)



# Compute LOO for each model
loo_weak <- loo(weak_prior_model)
```

```
Warning: Using only the first imputed data set. Please interpret the results with caution unt
il a more principled approach has been implemented.
Error in serialize(data, node$con) : error writing to connection
```

```
Error in serialize(data, node$con) : error writing to connection
```
