# Supplementary material for: Testing the role of online group-based supervision for local humanitarian workers following a crisis: A mixed-methods longitudinal study
Source: PLOS Glob Public Health. 2025 Aug 18;5(8):e0004635. doi: 10.1371/journal.pgph.0004635 (PMC12360539; doi:10.1371/journal.pgph.0004635)
Supplement: Attachment — Submitted filename: Response to Reviewers 24.07.25.docx [file pgph.0004635.s004.docx]

Dear Dr. Mowafi,

Thank you very much for giving us the opportunity to address the reviewers’ comments and feedback. We thank the reviewers for their insightful comments and suggestions to improve our paper. We addressed each comment and feedback point below, highlighting the changes in yellow in the track changes document and referencing the respective pages and lines in the Manuscript.

We believe that the revised revisions substantially improved our manuscript. We hope this version of the manuscript will be satisfactory to be considered for publication.

Best regards,

Dr. Gülşah Kurt

On behalf of the authors.

Review Comments to the Author

Reviewer #1: This manuscript presents a timely and important mixed-methods study investigating the stress-buffering role of online group-based supervision for Syrian mental health workers following the 2023 Türkiye-Syria earthquakes. The topic is highly relevant to global public health, particularly in humanitarian and disaster-affected settings. The mixed-methods approach is a strength, offering both quantitative evidence of the supervision program's effectiveness and qualitative insights into its key features. The authors have utilized a quasi-experimental design, taking advantage of a unique natural experiment. The findings suggest that post-earthquake supervision is associated with significant improvements in psychological distress and compassion satisfaction. While the study has significant strengths, there are areas where clarity, detail, and discussion could be enhanced to improve its impact and suitability for publication.

- **Reply:** We would like to thank the reviewer for their constructive evaluation of our study, highlighting the key strengths of the paper. We addressed each suggestion for improving the manuscript below.

-Title: The title is evocative and effectively captures the essence of the study's qualitative findings. However, the first part ("Everything changed, this was the constant thing that stayed the same.") could be integrated more smoothly with the descriptive part to maintain a professional academic tone. Consider rephrasing for conciseness while retaining its impact. Consider a more concise and direct title while retaining the essence of the mixed-methods approach and key findings.

- **Reply:** We updated the title as follows:

Testing the role of online group-based supervision for local humanitarian workers following a crisis: a mixed-methods longitudinal study

-Abstract: The abstract provides a good overview of the study. Ensure all quantitative results mentioned (e.g., specific changes in distress/satisfaction) are precisely stated and accompanied by their statistical significance.

- **Reply:** The abstract has been updated accordingly (highlighted in yellow below):

The 2023 Türkiye-Syria earthquakes caused widespread destruction, leaving millions without access to basic needs and services. Caring for Carers (C4C), an online group-based supervision program for Syrian mental health workers, started three months prior to the earthquakes. This study aimed to examine the role of this program in supporting the mental and occupational health of the participants following this crisis. A mixed-method longitudinal design was employed with Syrian mental health workers in Türkiye and Northwest Syria (N = 55, 41.8% female), including 16 months of survey data on psychological distress (Kessler-6) and compassion satisfaction (ProQOL), semi-structured interviews with supervisors (N = 10), and video recordings of supervision sessions (N = 9). A piecewise mixed-effects model within a Bayesian Hierarchical framework was used to assess changes in outcomes across three periods: the active control period (7 months), *pre-earthquake supervision period* (3 months), and *post-earthquake supervision period* (6 months). The thematic analysis was used to analyze the qualitative data from the interviews and supervision sessions. Quantitative findings showed a significant reduction in psychological distress and an increase in compassion satisfaction during *the post-earthquake supervision* *period* (b=-0.18, error=0.06, CrI = -0.29, -0.07, b = 0.26, error = 0.04, CrI =0.18, 0.35, respectively). These changes were significantly different from the active control period (b = 0.21, error= 0.05, CrI=0.11, 0.31, b = -0.37, error= 0.04, CrI = -0.45, -0.28, respectively) while no significant differences were observed between the active control and pre-earthquake supervision periods. Thematic analysis identified five key features of supervision as a safe space and two key functions as a source of emotional and practical support after the earthquakes. Overall, these findings provide initial evidence for both the protective and promotive role of supervision among Syrian mental health workers, highlighting the key mechanisms through which it may foster resilience and strength in humanitarian settings.

-Supervision Program (C4C): More details could be provided on the content of the supervision sessions. While it's mentioned that supervisors were given a handbook and participants presented case studies, elaborating on the typical topics, skills addressed, or therapeutic approaches discussed would strengthen the methodological description.

- **Reply:** We have expanded our description of the supervision program to include more details on the content and focus of the sessions. Please see the revised section below. **Page 8, Line 174-179 and Page 9 Line 180-185.**

The C4C supervision program was designed based on a supportive and reflective supervision modality to address the varied needs of participants in a group format. We aimed not to prescribe a specific supervision model but to allow flexibility in choosing how to facilitate the sessions. We provided supervisors with a handbook including contextual information, suggested session structures, and reflective prompts and examples. Each participant was asked to present at least one case study of their choice, and supervisors co-facilitated group discussion on this case. Case presentations typically focused on complex presentations of psychological problems related to traumatic experiences, loss and grief, family violence and parenting, and challenges in the therapeutic process, such as navigating cultural and gender norms and managing personal boundaries and self-care. Supervisors usually facilitated group discussions on accurate assessment and case formulation, as well as the use of evidence-based strategies such as cognitive-behavioral therapy techniques and exposure through demonstrations and role-plays. The groups also explored ways to address broader social needs related to prolonged conflict and forced displacement through culturally grounded community-based approaches. Additional skills covered included active listening, fostering non-judgmental practitioner responses, and emphasizing practitioner wellbeing and the recognition of effort and care, especially in complex cases where progress may be difficult to observe.

-Discuss attrition rates and how missing data were handled (e.g., were dropouts related to earthquake impacts?

- **Reply:** Data regarding the rate and ways of handling missing data is provided in detail under the Statistical Analysis Section (**Page 15, Line 324-334**) as below.

The average completion rate of surveys per time point was 41.95 (5.11), ranging from 55 to 33. Data were determined to be missing completely at random (MCAR) through the use of Little's MCAR test, Chi-Square = 752.896, DF = 727, Sig. = .246. We, therefore, imputed missing data in the outcome variables (compassion satisfaction and psychological distress). We used an intention-to-treat approach, including all cases in the analysis. Multiple imputation was conducted in Mplus (version 8.10) (Muthén & Muthén, 2019) using a regression model (where variables with missing data are regressed on variables without missing data) and analysis type basic. In Mplus, missing data imputation is conducted using Bayesian analysis which has been shown to provide robust imputation with multilevel longitudinal data (44). We imputed 10 datasets in Mplus (see supplementary material for the code) and then imported them into R for analysis.

We added additional information about the attrition rates from active control to the pre-earthquake and to the post-earthquake period to show that there were no significant differences between those who continued in the supervision program after the active control period and those who did not (**Page 17, Line 364-372**).

Among the 55 participants, 47 (85.45%) started the first supervision term in the program. Those who dropped out (N = 8) indicated several reasons for discontinuing, such as contract termination, changes in employment, declining to participate, or being unresponsive. There were no statistically significant differences between those who dropped out and those who continued in the program in terms of key demographic characteristics (e.g., gender (χ²(1) = 0.24, *p* = 0.63 and age (t (53) =1.59, *p* = 0.12) or their initial levels of psychological distress (*t*(53) = 0.50, *p* = 0.62) and compassion satisfaction (*t*(53) = 0.39, *p* = 0.70). 46 out of 47 (97.87%) continued the program after the earthquakes. One participant, unfortunately, passed away due to the earthquakes.

-The "stress-buffering" effect is compelling, but causality cannot be inferred due to the quasi-experimental design. Frame conclusions more cautiously.

- **Reply:** We agree with the reviewer that our design does not allow causal inferences for the observed effect of the supervision program. We have ensured that this limitation is clearly acknowledged in the manuscript as follows (**Page 32, Line 677-681**):

It is also important to note that the quasi-experimental design of the study does not allow us to draw causal conclusions. While our findings suggest a potential stress-buffering role of the supervision program, these results should be interpreted with caution and replicated in future studies using other designs such as randomized controlled trials.

-Contrast the study’s results with prior literature on supervision in non-humanitarian settings, and/or other humanitarian settings to highlight uniqueness.

**Reply:** We expanded the discussion of our results through a comparison with existing literature from both non-humanitarian and humanitarian settings. We believe that this expanded section (highlighted in yellow) has allowed us to more clearly position our findings within the literature and highlight their unique contributions. **Page 28, Line 589-594, 596-604 and Page 29, Line 605-606.**

While prior research documented the role of clinical supervision in reducing psychological distress and improving occupational outcomes, such as increasing job motivation and staff retention, both in low (16) and high resource settings (19), our study extends this literature by demonstrating that supervision may also play a stress-buffering role in the event of a large-scale humanitarian emergency. This is particularly relevant in pernicious and volatile settings where mental health workers are at a higher risk of experiencing multiple traumatic experiences and stressors. Existing studies in humanitarian settings have also emphasized the importance of supervision for both the well-being of humanitarian workers and the quality of service delivered (48). However, most of this research is either cross-sectional, showing the association between perceived supervisor support and outcomes (49), or focuses on the role of supervision in enhancing competence rather than well-being (50). More recently, studies in complex humanitarian settings (e.g., Ukraine, Afghanistan, Jordan, and Bangladesh) have examined the acceptability, effectiveness, and scalability of a multi-component supervision training program by assessing changes in knowledge, confidence, and psychological outcomes following training completion, rather than directly testing the impact of receiving ongoing supervision for humanitarian workers. Thus, the present study provides initial empirical evidence on the effectiveness of online, group-based supervision in supporting the mental health of humanitarian workers.

**Page 29, Line 619-623:**

The qualitative inquiry into the supervision sessions and the interviews with supervisors enabled us to explore the key features and functions of the supervision in-depth and complement the quantitative results. The findings highlighted the main role of supervision as being a safe and supportive space for practitioners where they discussed their personal and work-related challenges and received both emotional and practical support from their peers and supervisors. This aligns with extensive research showing the supportive function and practical and emotional benefits of supervision for mental health professionals (51,52).

-Strengthen Implications: Expand on the practical and policy implications of your findings, particularly for humanitarian organizations and mental health support programs.

- **Reply:** We thank the reviewer for this recommendation. We have expanded the implications section of the findings as follows **(Page 30, Line 646-650 and Page 31, Line 651-655):**

For humanitarian organizations, this means placing supervision at the centre of their MHPSS portfolio, not as an ad-hoc to trainings, but as an essential component of routine service delivery to ensure the provision of quality care that supports both mental health workers and service users. To enable an effective and sustained MHPSS response, supervision must be integrated into all programs with ongoing funding allocated throughout the implementation of MHPSS programs. These structural changes to funding, policy, and program design require a coordinated effort and strong commitment from multiple stakeholders, such as international agencies, donors, implementing organizations, and mental health workers to establish supervision as a standard practice that safeguards staff well-being and upholds the quality of MHPSS services.

Overall, this is a well-designed and executed study with important findings. Addressing these points will significantly enhance its clarity, rigor, and impact for publication in an academic journal.

- **Reply:** We would like to sincerely thank the reviewer for their constructive feedback and valuable suggestions to improve the manuscript. We are pleased to confirm that all comments and recommendations have been addressed in the revised version. We hope that this version will be suitable for publication.

Reviewer #2: This paper meets the criteria for publication in PLOS Global Public Health. The paper presents preliminary evidence for the use of an online supervision initiative to support the mental health workers providing care in aftermath of the Türkiye-Syria earthquakes. This is particularly important given the increase in frequency of natural disasters both in the region and internationally, which will require innovative and novel methods of providing and maintaining acute and chronic mental health care provisions. The use of mixed methods to present the data is appropriate with qualitative analyses being appropriate to further explore and contextualize quantitative data. They are also reproducible. Overall, the data and subsequent analyses support the claims that The Caring for Carers (C4C) project may improve psychological distress and compassion satisfaction among Syrian mental health workers in this context, albeit using a small sample size (n=55) to support these claims. The paper is written succinctly, although the abstract of the paper may benefit from simpler, more technical wording.

- **Reply:** We thank the reviewer for their constructive feedback and comment on the rigor and novelty of our study. Following the feedback related to the abstract, we revised it to enhance its clarity and academic tone as follows:

The 2023 Türkiye-Syria earthquakes caused widespread destruction, leaving millions without access to basic needs and services. Caring for Carers (C4C), an online group-based supervision program for Syrian mental health workers, started three months prior to the earthquakes. This study aimed to examine the role of this program in supporting the mental and occupational health of the participants following this crisis. A mixed-method longitudinal design was employed with Syrian mental health workers in Türkiye and Northwest Syria (N = 55, 41.8% female), including 16 months of survey data on psychological distress (Kessler-6) and compassion satisfaction (ProQOL), semi-structured interviews with supervisors (N = 10), and video recordings of supervision sessions (N = 9). A piecewise mixed-effects model within a Bayesian Hierarchical framework was used to assess changes in outcomes across three periods: the active control period (7 months), *pre-earthquake supervision period* (3 months), and *post-earthquake supervision period* (6 months). The thematic analysis was used to analyze the qualitative data from the interviews and supervision sessions. Quantitative findings showed a significant reduction in psychological distress and an increase in compassion satisfaction during *the post-earthquake supervision* *period* (b=-0.18, error=0.06, CrI = -0.29, -0.07, b = 0.26, error = 0.04, CrI =0.18, 0.35, respectively). These changes were significantly different from the active control period (b = 0.21, error= 0.05, CrI=0.11, 0.31, b = -0.37, error= 0.04, CrI = -0.45, -0.28, respectively) while no significant differences were observed between the active control and pre-earthquake supervision periods. Thematic analysis identified five key features of supervision as a safe space and two key functions as a source of emotional and practical support after the earthquakes. Overall, these findings provide initial evidence for both the protective and promotive role of supervision among Syrian mental health workers, highlighting the key mechanisms through which it may foster resilience and strength in humanitarian settings.
